# OMIP‐062: A 14‐Color, 16‐Antibody Panel for Immunophenotyping Human Innate Lymphoid, Myeloid and T Cells in Small Volumes of Whole Blood and Pediatric Airway Samples

**DOI:** 10.1002/cyto.a.23907

**Published:** 2019-10-21

**Authors:** Dawid Swieboda, Yanping Guo, Sophie Sagawe, Ryan S. Thwaites, Simon Nadel, Peter J.M. Openshaw, Fiona J. Culley

**Affiliations:** ^1^ National Heart and Lung Institute Imperial College London London UK; ^2^ Asthma UK Centre in Allergic Mechanisms of Asthma London UK; ^3^ Paediatric Intensive Care Unit St Mary's Hospital, Imperial College Healthcare NHS Trust London UK

## Purpose and Sample Types

This 14‐color, 16‐antibody OMIP was designed for enumeration of leukocyte responses in pediatric samples, where sample volumes and cell numbers can be very low. Leukocytes identified by this panel include all major members of the innate lymphoid cell (ILC) family (ILC1s, ILC2s, and ILC3s), natural killer cells (NK cells), granulocytes (neutrophils and eosinophils), T‐cells (CD4^+^ and CD8^+^), mucosal‐associated invariant T cells (MAIT cells) and NKT‐like cells. The protocol was optimized using small volumes of peripheral blood and validated in airway samples obtained from children (< 2 years of age) admitted to a pediatric intensive care unit (PICU). Given this backdrop, this OMIP is widely applicable to clinical research using low volume or paucicellular samples, such as studies of innate and adaptive immune responses in infants and children, with potential clinical application in diagnostics and monitoring of patients by pediatricians.

## Background

The immaturity of the immune system in early life renders infants vulnerable to infectious diseases, particularly those of the gastrointestinal and respiratory tracts. According to the World Health Organization, acute lower respiratory tract infections are the leading cause of death and hospitalization in children globally 1. How the immune system matures in early life is not fully understood. A better understanding of the nature of the immune and inflammatory response to infection in infants and why some infants develop disease could help to identify new clinical biomarkers, treatments, and prophylactics.

One of the major hurdles in the field of neonatal and pediatric immunology is obtaining relevant samples, which is limited by ethical restrictions on the volume and number of samples that can be taken. As a result, much of our understanding of cellular immunity in infants comes from studies of umbilical cord blood. Less is known about immunity in more mature infants, and less still is known about immunity to infection at mucosal sites, such as the lung 2, 3, 4. Moreover, the use of invasive procedures to obtain, for example, lower airway samples cannot be justified in healthy infants. It is important, therefore, that techniques are developed that facilitate the characterization of the immune response in small‐volume and paucicellular samples, particularly from sites of infection where sampling is limited. In designing this OMIP 5 we focused on developing a panel to enumerate the innate lymphoid cell (ILC), granulocyte and T‐cell responses in infants, particularly those with a lung infection (Table 1). To reflect the challenging situation of limited amounts of blood and airway samples from infants we optimized this panel using a small volume (300 μl) of adult peripheral blood. Pitoiset et al. (2018) were able to detect regulatory T cells (Tregs) in as little as 60 μl of whole blood from children 6 and others have demonstrated the feasibility of detecting T cell subsets and Type 2 ILCs within pediatric airway samples 7, 8.

**Table 1:** Summary table

Purpose	Myeloid and innate/adaptive lymphoid comprehensive immunophenotyping
Cell types	Whole peripheral blood, cord blood, PBMCs, nasal aspirate, and tracheal aspirate samples
Species	Human
Cross‐reference	OMIP‐55, OMIP‐007, OMIP‐27, OMIP‐029, OMIP‐038, OMIP‐039

The recently discovered ILCs have been implicated in playing a pivotal role in immune responses to viral and bacterial pathogens, and they are particularly abundant at mucosal sites 9, 10, 11. There are three main subsets of ILC; ILC1s, ILC2s, and ILC3s, which may mirror CD4^+^ T helper 1 (Th1), Th2, and Th17 subsets, whereas NK cells may complement the cytokine profile and function of CD8^+^ T cells 12. ILCs comprise only 0.1 to 0.01% of lymphocytes in adult blood 13 and most published methods for detection of ILCs are optimized on relatively large volumes of peripheral blood, however, since ILCs are relatively abundant in children we anticipated relatively high frequencies in small volume pediatric samples 8, 14, 15. Immunophenotyping ILCs can be challenging as they are defined as lineage negative lymphocytes, which lack specific markers, including CD3 (T cells), CD19 (B cells), CD34 (progenitor cells), FcεR1α (mast cells) and CD1a, CD123, BDCA2 (plasmacytoid dendritic cells, pDC) 15, 16. Accordingly, while designing this panel we made sure that a very stringent gating strategy was utilized to obtain a pure ILC subset. We used biaxial gating to delineate ILCs as lineage negative, CD127 (IL‐7 receptor‐α) positive, live lymphocytes. We distinguished ILC subsets using surface expression of prostaglandin D_2_ receptor chemoattractant receptor‐homologous molecule expressed on Th2 cells (CRTH2) and CD117 (c‐Kit) 8, 10, 17. In designing this OMIP other components of the cellular immune response were included, which gives this panel broad applicability to other pediatric clinical studies. These were myeloid cells (neutrophils, eosinophils), invariant T cells (mucosal invariant T cells (MAITs) and NKT‐like cells) and adaptive T cells (CD4^+^ and CD8^+^). By immunophenotyping whole blood, rather than peripheral blood mononuclear cells (PBMC), we reduced the processing time and retained granulocyte populations. All fluorochrome‐conjugated antibodies were titrated during panel optimization and are listed in Table 2.

**Table 2:** Antibodies used in the optimized multicolor immunofluorescence panel (OMIP‐062)

Antibody	Fluorochrome	Ab Clone	Purpose
CD127 (IL‐7Rα)	BV421	A019D5	ILCs
CD14^a^	BV510	63D3	Lineage
CD19^a^	BV510	HIB19	Lineage
FcεRIα^a^	BV510	AER‐37 (CRA‐1)	Lineage
CD123^a^	BV510	6H6	Lineage
CD4	BV605	RPA‐T4	CD4^+^ T cells
CD16	BV650	3G8	NK cells/neutrophils
CD8	BV711	SK1	CD8^+^ T cells
TCR Vα7.2	BV785	3C10	MAIT cells
CD45	FITC	HI30	Leukocytes
CD117 (c‐kit)	PerCP‐Cy5.5	A3C6E2	ILC3
CD3^b^	PE	OKT3	T cells
CD161	PE‐Dazzle	HP‐3G10	MAIT cells
CD56 (NCAM)^b^	PE‐Cy7	5.1H11	NK/NKT‐like cells
CD294 (CRTH2)	AF647	BM16	ILC2/Th2/Tc2 subsets
CD66b	AF700	G10F5	Eosinophils
Live/Dead	Near IR‐fluorescent reactive dye	n/a	Viability

a Lineage cocktail includes the following antibodies: CD14, CD19, CD123, FcεR1α.

b Antibodies used to define the lineage negative population, but not part of the lineage cocktail.

**Figure 1:**
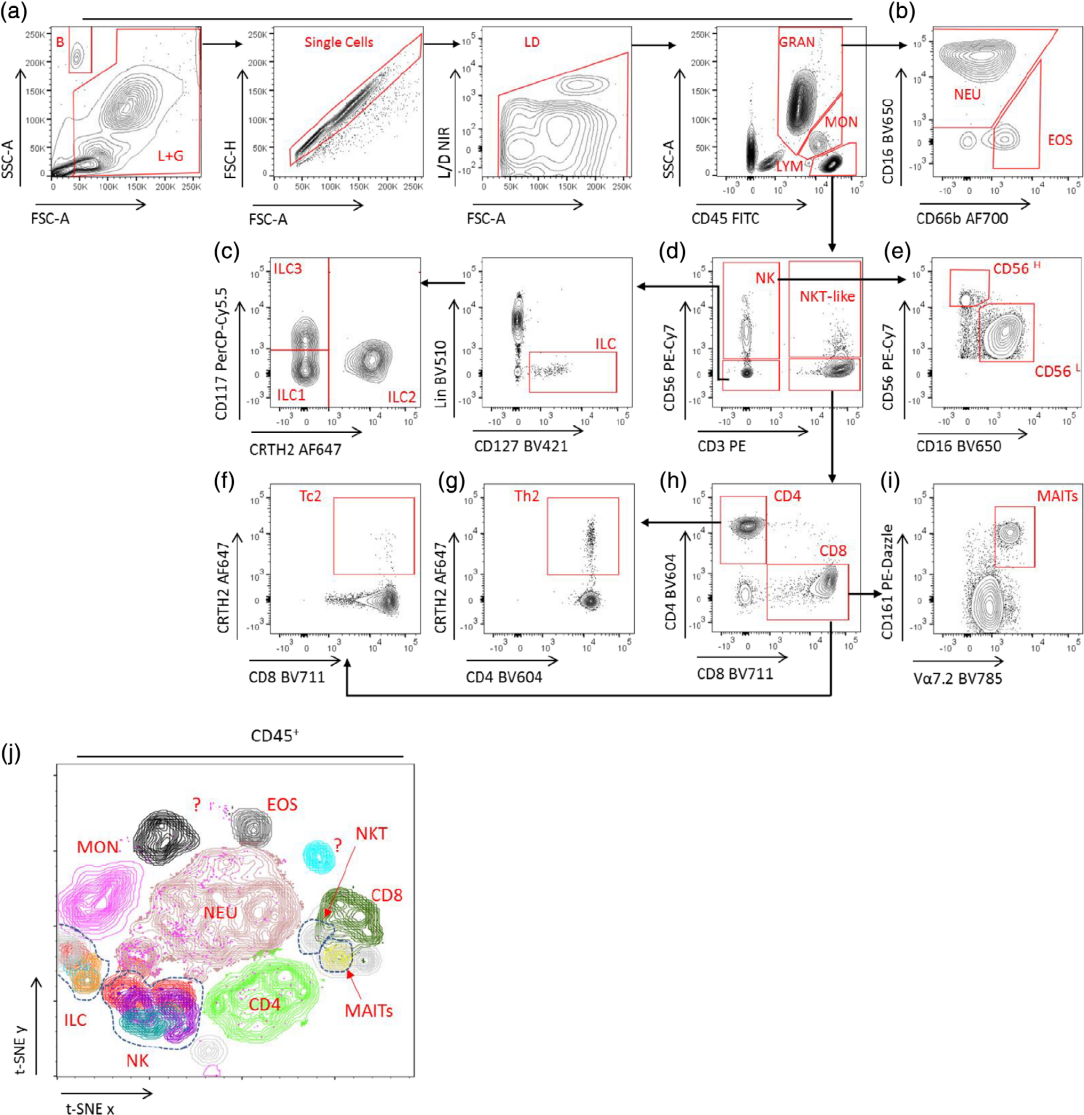
Example gating strategy and t‐SNE analysis for visualization of ILCs (including NK cells), granulocytes, MAIT, NKT‐like cells, and other T cell populations in pediatric clinical samples. Abbreviations: B, counting beads; L + G, lymphocyte and granulocyte gate; LD, live/dead; GRAN, granulocytes; MON, monocytes; LYM, lymphocytes; NEU, neutrophils; EOS, eosinophils; ILC, innate lymphoid cells; MAIT, mucosal‐associated invariant T cells; NK, natural killer cells; NKT‐like, natural killer T like cells; ?, Unidentified populations. [Color figure can be viewed at http://wileyonlinelibrary.com]

Cells were delineated using the following gating strategy: FSC/SSC, single cells, live cells. Granulocytes, monocytes, and lymphocytes were gated using FSC and the CD45 cell marker (Fig. 1A). Within the granulocyte population, neutrophils were defined as CD16^H^CD66b^+/−^ and eosinophils as CD16^+/‐^CD66b^+^ (Fig. 1B). Within the lymphocyte gate, the markers CD56 and CD3 were used to identify NK cells (CD56^+^CD3^−^), conventional T lymphocytes (CD3^+^CD56^−^) and NKT‐like cells (CD56^+^CD3^+^) (Fig. 1D) 18, 19, 20. NK cells were further subdivided into CD56^H^CD16^L^ and CD56^L^CD16^H^ populations according to the CD56 and CD16 density (Fig. 1E). T lymphocytes (CD3^+^CD56^−^) were segregated into CD4^+^ or CD8^+^ T cells (Fig. 1H). Th2 and Tc2 cells were defined within the CD4^+^ and CD8^+^ T cell subsets, respectively, using the CRTH2 surface marker, as these Type‐2 cytokine‐producing cells express high levels of CRTH2 8, 21 (Fig. 1F,G). Within CD8^+^ T cells, we also identified MAIT cells. MAIT cells are invariant T lymphocytes which respond to riboflavin metabolites in the context of the MHC class I related molecule MR1 22, 23. MAIT cells were identified within the CD3^+^CD56^−^CD8^+^ gate as CD161^H^Vα7.2^+^cells (Fig. 1I). NKT cells are conventionally described as CD3^+^CD56^+^ lymphocytes 24, 25, however, the use of these markers alone defines a heterogeneous population of lymphocytes, which we designate here as “NKT‐like” cells. CD56 can be expressed by many CD3^+^ lymphocyte subpopulations, including invariant NKT cells (iNKT cells), and some γδ T cells and MAIT cells (which have previously been described as mucosal NKT cells) 26, 27. Strategies to define these subpopulations are discussed further in the Supporting Information (Part B). Finally, ILCs were defined using the following gating strategy: live, single, CD45^+^ lymphocytes, lineage (CD3, CD56, CD14, CD19, CD123, FcεR1α) negative, and CD127^+^ cells (Fig. 1C); ILC1s were defined as CD117^−^CRTH2^−^, ILC2s as CRTH2^+^ CD117^int^, and ILC3s as CD117^+^CRTH2^−^ 15, 28.

To further analyze different populations of cells in this OMIP, unbiased high dimensional stoichiometry (t‐SNE) analysis was performed on live, single, CD45^+^ cells incorporating the lymphocyte, granulocyte, and monocyte gates (Fig. 1J). In this example, t‐SNE not only revealed that all the populations of cells segregated according to the gating strategy illustrated in Figure 1A–I but additionally discriminated distinct or poorly defined populations. Therefore, this 14‐color flow panel can be used to delineate other unknown populations using high‐dimensional data visualization techniques 29.

Following optimization on adult peripheral blood, we confirmed that the panel was suitable for staining umbilical cord blood, and peripheral blood, tracheal aspirate, and nasal aspirate samples from infants. The inclusion of CD45 in the panel allowed us to separate leukocytes from structural cells, such as epithelial cells, found in airway samples. Concentrations of antibodies were kept the same for analysis of different samples as they gave the same optimal separation between positive and negative populations; however, the protocol for staining airway samples was more methodically complicated and included the use of Fc block. Counting beads were included to allow absolute counts of cell numbers. Detailed information on the panel development and optimization can be found in the Supporting Information (Part B).

## Human Samples

All blood and airway samples were collected from subjects after gaining written consent. Some samples were collected as a part of the Early Life Lung Infection (ELLI) and RSV‐SAM studies, at St. Mary's Hospital, London. The use of all human tissue samples was approved by the Health Research Authority (HRA) and Health and Care Research Wales (HCRW) Ethics Committee (REC numbers 18/LO/1570; 15/WM/0343 and 13/LO/1712) and in accordance with the Declaration of Helsinki 1964.

## Similarity to Other OMIPs

OMIP‐55 cross‐references to the ILC immunophenotyping; OMIP‐007, OMIP‐027, and OMIP‐029, OMIP‐38 and OMIP‐039 include phenotypic analysis of NK cells.

## Conflict of interest

The authors have no conflicts of interest to declare.

## Funding

Research Support: Asthma UK Centre in Allergic Mechanisms of Asthma, Imperial College London; Grant number: AUK‐BC‐2015‐01; National Institute for Health Research (NIHR) Biomedical Research Centre (BRC) based at Imperial College Healthcare NHS Trust and Imperial College London; Grant numbers: RDA02 and P82570, The Wellcome Trust; Grant number: 109008/Z/15/A.

## Supporting information


**Appendix S1**: Supporting Information

**MIFlowCyt**: MIFlowCyt‐Compliant Items
