# Supplementary material for: OMIP‐062: A 14‐Color, 16‐Antibody Panel for Immunophenotyping Human Innate Lymphoid, Myeloid and T Cells in Small Volumes of Whole Blood and Pediatric Airway Samples
Source: Cytometry A. 2019 Oct 21;95(12):1231–5. doi: 10.1002/cyto.a.23907 (PMC6972618; doi:10.1002/cyto.a.23907)
Supplement: Supplementary file 1 — Appendix S1: Supporting Information [file CYTO-95-1231-s001.docx]

A 14-color, 16-antibody panel for immunophenotyping human innate lymphoid, myeloid and T cells in small volumes of whole blood and pediatric airway samples

Swieboda *et al.* (2019)

*ONLINE MATERIAL*

**Panel design and development**

We aimed to design a panel that would allow us to assess innate and adaptive immune responses in small volumes of peripheral blood and paucicellular samples. We designed a 14-color, 16- parameter panel using recently published literature on cell surface markers for different components of the immune response (1–9). Innate immunity is the first line of defense against a myriad of pathogens (10,11) and is particularly important in early childhood due to immature adaptive responses (12,13). In this panel, we placed particular emphasis on identification of lineage negative (10), CD127 positive innate lymphoid cells (ILCs) but we also comprehensively evaluated components of the T cell response, such as CD4^+^ (including Th2s), CD8^+^ (including Tc2s), and invariant T cells (MAITs and NKTs). Our panel also allows the simultaneous identification of eosinophil and neutrophil populations.

*Panel design strategy*

To establish protocols for use with samples with low cell numbers, 300 µL of peripheral blood from healthy adult volunteers was utilized for panel optimization **(Figure 1, Part A).**

All fluorochrome-conjugated antibodies used in this OMIP are commercially available (**Online Table 2**). During the process of designing this panel, we used the principle that fluorochromes with the brightest staining index would be used for surface markers with the lowest antigen density or on the rarest cell populations. For antigens that are highly expressed, dimmer fluorochromes were more suitable; however, some level of compromise was required due to the flow cytometer configuration, and the commercial availability and affordability of fluorochromes and antibodies. We also placed emphasis on the reduction of spectral overlap by placing antigens that co-localise such as CD45 (clone HI30, FITC), CD3 (clone OKT3, PE), CD4 (clone RPA-T4, BV605) and CD8 (clone SK1, BV711) on different lasers. In order to increase the emission range from lasers, we used tandem dyes such as PerCP-Cy5.5 for CD117 (clone A3C6E2), PE-Dazzle for CD161 (clone HP-3G10) and PE-Cy7 for CD56 (clone 5.1H11).

All fluorochrome-conjugated antibodies and tandem dyes, in particular, are prone to degradation by light or chemicals used during sample handling that might lead to false-positive data. To address the problem of fluorochrome degradation, we determined the impact of time (1, 2 or 3 days) between staining and analysis, and different fixative reagents (fixation buffer after red blood cell (RBC) lysis or combined RBC lysis/fixation solution) on fluorochrome brightness. Time and different protocols of RBC lysis and fixing had no impact on the percentage and the total number of live, single cells or on granulocytes, ILCs populations and other cell subtypes up to three days post-staining (data not shown, available upon request).

Non-optimal concentrations of antibody might increase non-specific binding or reduce the sensitivity of the measurement. Therefore, all fluorochrome-conjugated antibodies were titrated to determine the best signal-to-noise ratio (**Online Figure 1, 2 and 3**). Negative control with no antibody added was included for all titrations. The stain index (SI) was calculated as follows:

$$SI = \frac{(MFI positive population-MFI negative population)}{2 x SD MFI negative population}$$

Where MFI = geometric mean fluorescence intensity, SD = standard deviation.

Once established, the concentrations of antibodies used were kept the same throughout experiments to give optimal separation between positive and negative populations.

Dead cells can be a source of false positives and non-specific binding and very often cannot be removed by FSC and SSC gating. In this panel, we used LIVE/DEAD™ Fixable Near-IR Dead Cell Stain Kit (Thermofisher, Cat. No. L-10119), which provided a bright signal and a great level of separation upon excitation with a red laser (633 nm). The Live/Dead dye was titrated in a range of dilutions (1:100, 1:250, 1:500 and 1:1000) to determine the best concentration for separation of live and dead populations (**Online Figure 4**).

During the development of this panel, we used fluorescence minus one (FMO) controls for each of the antibodies to determine fluorescence spread, increase sensitivity and accurately describe gating boundaries (**Online Figure 5**). We found that FMO controls were not necessary for each experiment as many cell populations were clearly defined on the flow plots. Other gates, however, are more difficult to place, such as CD117 which defines ILC subpopulations, and we recommend the regular use of FMO controls if sufficient cells are available.

As shown in **Figure 1 (Part A)** we gated the initial population on FSC-A/SSC-A, removed doublets with FSC-A/FSC-H and selected live cells using negative staining, as those unstained by the Fixable Near-IR Dead Cell Stain. We were able to identify the populations of granulocytes, monocytes, and lymphocytes using SSC-A and the CD45 (FITC, clone HI30) cell marker. CD45 is highly expressed on all leukocytes in human peripheral blood and cord blood (14). The inclusion of CD45 in this panel is mandatory when working with airway samples such as nasopharangeal aspirate (NPA) and tracheal aspirate (TA), to exclude non-hematopoietic cells such as epithelial cells. CD45-positive lymphocytes were further gated with CD56 (PE-Cy7, clone 5.1H11) and CD3 (PE, clone OKT3) to select NK and NKT-like cells (CD56^+^CD3^-^ and CD56^+^CD3^+^, respectively) (15,16). We also tested CD56 staining using clone 5.1H11 conjugated to BV650, but no significant advantage was noted compared to the PE-Cy7 antibody used in the final panel (**Online Figure 3**).

Within the CD3^+^CD56^-^ gate, we identified CD4^+^ and CD8^+^ T cells. CRTH2 clone BM16 on AF647 allowed discrimination of type 2 cells among CD3^+^CD8^+^ (Tc2 cells) and CD3^+^CD4^+^ (Th2 cells) T lymphocytes as described (17,18). Mucosal-associated invariant T (MAIT) lymphocytes are strongly associated with mucosal surfaces such as the lungs and recognize non-peptide antigens such as riboflavin metabolites (19,20). The α chain of the invariant T cell receptor expressed by these cells is comprised of the Vα7.2 and Jα33 segments. These cells also express high levels of CD161. To delineate these cells we used CD161 on PE-Dazzle (clone HP-3G10) and Vα7.2 on BV785 (clone 3C10) within the CD3^+^CD8^+^ T lymphocyte population.

CD3 and CD56 are conventional markers for defining NKT cells, however, CD3^+^ CD56^+^ NKT-like cells are a heterogeneous population, which includes invariant NKT cells, some γδ T cells and MAIT cells, although not all MAIT cells are CD56^+^. Using our OMIP, we can identify CD4^+^ and CD8^+^ subsets within the NKT-like cells gate and CD56^+^ MAIT cells as CD3^+^ CD56^+^ CD8^+^ CD161^H^ Vα7.2^+^ cells (**Online Figure 6**) (21). An alternative gating strategy is shown in **Online Figure 7** in which total MAIT cells can be identified within CD3^+^CD8^+^ lymphocytes and removed from remaining populations. MAIT cells (both CD56^+^ and CD56^-^) are first identified as CD3^+^CD8^+^CD161^H^ Vα7.2^+^ lymphocytes, and populations of conventional CD8^+^ and CD8+ NKT-like cells are gated for within the remaining non-MAIT cells.

CD16 on BV650 with CD66b on AF700 allowed us to delineate neutrophils and eosinophils (CD16^H^CD66b^+/-^and CD16^+/-^CD66b^+^, respectively) within the CD45^+^ granulocyte gate in whole blood. The inclusion of CD16 in the OMIP also allows for the identification of subpopulations of NK cells based on their levels of CD56 and CD16 expression (**Figure 1, Part A**). Furthermore, this panel also allows enumeration of the subset of NK cells that express CD127 (not shown) (22).

ILCs were defined using the following gating strategy: live, single, CD45+ lymphocytes, lineage (CD3, CD56, CD14, CD19, CD123, FcεR1α) negative and CD127+ cells; ILC1s were defined as CD117−CRTH2−, ILC2s as CRTH2+ CD117int and ILC3s as CD117+CRTH2− . For ILC identification among CD3^-^CD56^-^ lymphocytes, clone A019D5 of CD127 on BV421 and the lineage cocktail on BV510 (CD14 clone 63D3, CD19 clone HIB19, FcεRIα clone AER-37 (CRA-1) and CD123 clone 6H6) provided good discrimination. Based on published data, we selected CRTH2 (prostaglandin D_2_ receptor) clone BM16 on AF647 and CD117 (c-Kit) clone A3C6E2 on PerCP-Cy5.5 for identification of different subsets of ILCs (3,17,23). As part of the panel optimization, we tested alternative clones for these markers. No alternative clones are widely available for CRTH2. We tested clone YB5.B8 for CD117 and found a lower level of discrimination of ILC3 compared to A3C6E2 (**Online Figures 2 and 8)**. We also compared clone HIL-7R-M21 for CD127 staining. Used at equal concentration to clone A019D5 we found comparable staining and sensitivity (**Online Figures 2 and 8)**. Different ILC populations identified using our gating strategy express different levels of CD127, as previously described (14). Furthermore, the inclusion of CD161 in the OMIP (clone HP-3G10 on PE-Dazzle) demonstrates different levels of CD161 expression on subsets of ILC, and can additionally be used to aid subset identification (**Online Figure 9**) (14).

*Data acquisition*

A BD 5-laser-LSRFortessa^™^ with the filter configuration shown (**Online Table 1**) was used throughout. Daily quality control and monitoring of the instrument was performed by running cytometry setting and tracking (CST) beads. Standard procedures were followed for setting up and shutting down the cytometer.

The PMT voltage of each parameter was optimized following these basic guidelines: positive populations are on scale; for compensation controls, positive signals detected from the actual PMT is higher than the overlapping signals detected in other parameters; the resolution of negative and positive populations are optimized, especially for those dim fluorochromes and weakly expressed markers. Once optimized, voltages were kept the same between experiments and no manual modifications of PMT values were performed. Single-stained compensation beads were run with each experiment and compensation was calculated using BD Diva software (**Online Figure 10**). In addition, to confirm the stability of the flow cytometer and the settings used, CST beads were run using the experimental settings before each experiment.

CountBright™ absolute counting beads were added to experimental samples and bead count and cell count were determined using FlowJoV10 software. The total number of cells was determined according to the manufacturer’s instructions.

*Panel adaptability*

This panel was optimized on a 5-laser-LSRFortessa^™^ cytometer with 6 PMTs on violet laser 405 nm, 3 PMTs on blue laser 488 nm, 4 PMTs on yellow/green 561 nm laser and 3 PMTs on 633 nm red laser (**Online Table 1**). We used 14 detectors out of 16 due to the limitations of compensation between channels: 6 markers at 405 nm, 2 markers at 488 nm, 3 markers at 561 nm and 3 markers at 633 nm (including Live/Dead staining). When designing the panel we intentionally excluded the UV laser in order to be applicable to a greater number of cytometers; however, given the recent increase in the availability of UV dyes and compatible cytometers, we believe that this provides additional opportunities for this panel to be expanded on instruments that include this laser.

We have modified the panel to describe the cytokine profile of lymphocytes within peripheral blood mononuclear cells (PBMCs). Some markers were removed (e.g. CD161, CD16, CD66b and Vα7.2) and others added, such as: IL-13 (PE-Cy7, JES10-5A2, Biolegend), IL-17A (AF700, BL168, Biolegend), IL-22 (FITC, clone 2G12A41, Biolegend), IFN-γ (BUV395, cloneB27, BD) which were used for intracellular staining following phorbol 12-myristate 13-acetate (PMA) and ionomycin stimulation in the presence of BD GolgiPlug™, cell fixation and permeabilization. All antibodies were titrated to determine the best signal and FMO controls used (data not shown). The removal of granulocyte markers in this particular application was justified, as the process of PBMC isolation removes granulocytes.

This panel could also be modified by the addition of antibodies to surface markers that allow further discrimination of innate lymphocyte subsets. For example, the Vα24Vβ11 TCR is characteristic of invariant NKT cells, which can also be identified using a CD1d tetramer, and Vδ1 and Vδ2 TCR usage identifies subsets of γδ T cells (OMIP 19 & 20) (24,25). CD57 can be used to identify the maturation status of NK cells. The removal of CD14 from the lineage cocktail onto an independent channel would allow the discrimination of monocyte subsets based on their CD14 and CD16 expression (OMIP 23) (26).

**Protocol optimization**

We tested a number of different processing and preservation protocols on blood and airway samples as part of our optimization of this panel (**Online Figures 11 and 12)**. Pediatric samples are ethically difficult to obtain and very often paucicellular. We optimized this panel using 300 μL of peripheral blood. Others have successfully used as little as 60μL of whole blood from pediatric patients to detect regulatory T cells (27); however, regulatory T cells comprise 1-5% of circulating CD4+ T cells, whereas Lin^-^ CD127^+^ ILCs represent only between 0.1 and 0.01% of lymphocytes in the periphery (1). We aimed to create a protocol that reduced the processing time, minimized the loss of viable cells and maintained the quality of the data. Many human studies utilize PBMCs, which involves time-consuming isolation steps and the loss of granulocyte populations (28). We found that immunostaining of whole blood prior to simultaneous RBC lysis and fixation (**Staining Protocol A**) saves processing time and will reduce the likelihood of variability due to cell death and loss of cell populations. We found no advantage to RBC lysis prior to staining (data not shown, **Online Figure 11)**.

When immunophenotyping whole blood, it is common practice not to use Fc-block due to the high level of immunoglobulins in the serum. Fc-receptors play an important role in immune responses and are detected on many hematopoietic cells, such as neutrophils, macrophages, eosinophils and NK cells (29). We compared staining of RBC-lysed whole blood with or without the use of Fc block and saw little difference in staining of our lymphocyte populations (data not shown). Nevertheless, we do recommend the use of Fc-receptor blockade as good practice when staining RBC-lysed blood, PBMC and airway samples (**Staining Protocol B**). Once optimized, we tested the panel on neonatal cord blood samples, adult and pediatric PBMC and pediatric airway samples, to confirm its suitability (**Online Figure 13**).

We tested different methods for preserving whole blood (**Online Figure 11**) and airway samples (**Online Figure 12**). We aimed to establish a protocol that would allow us to store samples prior to staining. This would have allowed us to stain and analyze multiple samples simultaneously, reducing technical variation. In our hands, however, freezing whole blood as a method of preservation was unsatisfactory. The recovery of functional, live cells was low with low numbers of granulocytes (**Online Figures 14**), which are lost during cryopreservation. Only the use of large volumes of purified granulocytes (up to 400mL of donated peripheral blood, buffy coats), and fixing them with 1 % PFA after thawing, has been reported to prevent this process (30). Similarly, freezing airway samples was not successful. We found that the populations of cells affected the most by freezing were lymphocytes, including ILCs, and to a lesser extent granulocytes (**Online Figures 12 and 15**). Although a very important tool in studying immunology, cryopreservation can induce significant changes in the number and viability of cells.

Subsequently, we evaluated the preservation of blood and airways samples (TA) using the stabilizing reagent TransFix™ (**Online Figures 11, 12, 16 and 17**). According to the published literature and the manufacturer’s instructions, TransFix™-treated whole blood remains stable for up to 14 days through the blockade of all apoptotic pathways and seems well-suited for short-term storage of samples (31). In our hands, TransFix™ preserved most surface antigens well (granulocytes, NK/NKT and MAIT cells). The biggest effect was observed on ILCs as a time-dependent decrease in CRTH2 staining was observed (**Online Figure 17**) when cells were stored in Transfix™ prior to staining. Similarly, TransFix™-treated TA samples were not well preserved with reduced lymphocyte and ILC populations (**Online Figure 16**). The significance of this finding is unclear, however, a progressive time-dependent necrosis-like behavior has been previously reported when working with TransFix™-stabilized granulocytes using transmission electron microscopy (31).

*Staining Materials*

Commercial and in-house reagents and materials were used (**Online Table 2 and 3**).

*Blood sampling, processing and storage*

For blood samples, we recommend staining in whole blood followed by fixation and lysis (**Online Figure 11; Staining Protocol A**). Blood from consenting healthy adult donors (typically 5-10 mL) was taken by venipuncture using a 23-gauge winged butterfly needle with a vacutainer system into 10 ml EDTA coated tubes (BD Biosciences, UK). Blood obtained from infants was typically 0.5-2.5ml. During optimization, we compared different protocols using adult blood. Blood was either processed fresh, stabilized using TransFix™ according to the manufacturer’s instructions, or frozen (**Online Figure 11)**. To freeze, blood was mixed with an equal volume of freezing medium (FM, 90% FCS, 10% DMSO). After mixing by inverting, samples were placed in a Nalgene™ Mr Frosty container for slow freezing to -80^o^C. After 2 days, cells were transferred into liquid nitrogen for long-term storage. During optimization we also compared RBC lysis (RBC lysis buffer, Biolegend) prior to staining and fixation (Fixation Buffer, Biolegend), with staining in whole blood followed by simultaneous lysis and fixation (RBC Lysis/Fixation Solution, Biolegend). Adult and cord blood PBMCs were isolated using density centrifugation on Lymphopure™ (BioLegend, UK) according to the manufacturer’s protocol. Cells were cryopreserved or used immediately (32). When staining blood samples in which RBC have been lysed, frozen blood or PBMC, we recommend using **Staining Protocol B**.

*Airway sampling, processing and storage*

For airway samples, we recommend staining airway cells fresh followed by fixation (**Online Figure 12; Staining Protocol B)**. NPA and TA were obtained as part of routine clinical care of infants on the Paediatric Intensive Care Unit at St Mary’s Hospital using previously optimized techniques (33). Collected samples were processed within 3 hours. The cellular component of airway samples was obtained by centrifugation. During optimization we compared different protocols. Airway cells were processed fresh, after stabilization with TransFix™ according to the manufacturer’s instructions, or after freezing in FM (**Online Figure 12**). Cells for freezing were mixed and placed in a Nalgene™ Mr Frosty container for slow freezing to -80^o^C. After 2 days, cells were transferred into liquid nitrogen for long-term storage.

**Staining procedures**

The procedures should be performed in a Class II laminar hood and according to a local risk assessment.

**Staining Protocol A: For flow cytometry on fresh whole blood**

Note 1: Whole blood (WB) staining is performed in flow cytometry tubes.

Note 2: Whole blood should be gently mixed in the collection tube before staining (up to 30 minutes on a roller).

1. Carefully aliquot 300 µL of WB into the flow cytometry tube.
2. Add the antibody cocktail and Live/Dead reagent according to the dilutions shown in **Online Table 2** (in our experience the antibody cocktail can be prepared up to 4 weeks in advance without the antibodies changing their properties).
3. Vortex and incubate at room temperature (RT) for 30 minutes (protect from light).
4. Lyse the RBC and fix the sample by adding 2 mL of RBC lysis/fixation solution (prepared according to the manufacturer’s instructions).
5. Vortex and incubate at RT for 5-10 minutes.
6. Check the level of RBC lysis under the light.
7. Centrifuge for 5 minutes, 800 x *g* at RT.
8. Discard the supernatant (if the level of RBC lysis is too low, the lysis step can be repeated but the yield of live cells may be reduced).
9. Resuspend the final sample in 370 µL of cell staining buffer. Samples can be stored in the dark, at 4^o^C until acquisition (no more than 2-3 days later). Just before acquisition counting beads are added up to 400 µL.

**Staining Protocol B: For flow cytometry on airway samples, PBMC, red-cell-lysed blood and frozen whole blood**

Note 1: Start with a single cell suspension. Mechanical disruption with a syringe and a needle can be performed when samples are mucus- or cell-rich.

Note 2: Count cells on Multicounting10™ Cell counting chamber slides using trypan blue.

Note 3: Plate 1-2.5 x 10^6^ cells per well in a V-bottomed 96-well plate for staining. Cells are transferred to flow cytometry tubes for acquisition.

Stage 1: Fc-blocking procedure

1. Spin cells down at 800 x *g* for 5 minutes at 4^o^C.
2. Remove supernatant and add 150 µL staining buffer.
3. Repeat Step 1.
4. Remove the supernatant and add 100μL Human TruStain FcX™ - Fc Block diluted 1:50 in cell staining buffer for 10 minutes at 4°C.
5. Wash cells by adding 150 µL PBS on top of the Fc-block and centrifuge at 800 x *g* for 5 minutes (‘’half’’ wash or “dirty” wash).
6. Remove the supernatant and wash by addition of 200μL PBS followed by centrifugation at 800 x *g* for 5 minutes and removal of the supernatant.

Stage 2: Surface and Live/Dead staining

1. Add 100µL per well of the antibody cocktail and fixable Live/Dead dye at the appropriate concentrations in PBS (**Online Table 2**).
2. Incubate for 30 minutes on ice or at 4^o^C in the dark.
3. Wash cells by adding 150 µL cell staining buffer on top of the staining cocktail and centrifuge at 800 x *g* for 5 minutes (‘’half’’ wash or “dirty” wash).

Stage 3: Fixation procedure

1. Remove the supernatant and wash cells by the addition of 200 µL cell staining buffer followed by centrifugation at 800 x *g* for 5 minutes. Remove the supernatant.
2. Resuspend cells in 100 µL of fresh fixation buffer.
3. Incubate in the dark for 30 minutes at 4^o^C, or at room temperature for 10-20 minutes.
4. Wash cells with 150 µL cell staining buffer on top of the fixation buffer and centrifuge at 800 x *g* for 5 minutes (‘’half’’ wash or “dirty” wash).
5. Wash cells again with 200 µL cell staining buffer, resuspend in 200 µL cell staining buffer, transfer to flow cytometry tubes and add 170 µL of staining buffer. Samples can be stored in the dark, at 4^o^C until acquisition (for no more than 2-3 days) at which point counting beads are added up to 400 µL.

**Cross-reference with related panels**

OMIPs related to work in this publication include OMIP-007, OMIP-027, OMIP-029 and OMIP-039 for NK and NKT cell analysis; OMIP-038 for innate responses, OMIP-55 for ILC identification.

**Data analysis**

Data were analyzed using FloJoV10 software (FlowJo, USA).

**References**

1. Hazenberg MD, Spits H. Review Article Human innate lymphoid cells. Blood.;124:700–10 (2014)
2. Klose, C. S. N. & Artis, D. Innate lymphoid cells as regulators of immunity, inflammation and tissue homeostasis. Nat. Immunol. 17, 765–74 (2016).
3. Dhariwal, J. et al. Mucosal Type 2 Innate Lymphoid Cells Are a Key Component of the Allergic Response to Aeroallergens. Am. J. Respir. Crit. Care Med. 195, 1586–1596 (2017).
4. Bal, S. M. et al. IL-1β, IL-4 and IL-12 control the fate of group 2 innate lymphoid cells in human airway inflammation in the lungs. Nat. Immunol. 17, 636–645 (2016).
5. Björklund, Å. K. et al. The heterogeneity of human CD127+ innate lymphoid cells revealed by single-cell RNA sequencing. Nat. Immunol. 17, 451–460 (2016).
6. Lakschevitz, F. S. et al. Identification of neutrophil surface marker changes in health and inflammation using high-throughput screening flow cytometry. Exp. Cell Res. 342, 200–209 (2016).
7. Hogan, S. P. et al. Eosinophils: Biological Properties and Role in Health and Disease. Clinical & Experimental Allergy 38, (2008).
8. Romagnani, S. T-cell subsets (Th1 versus Th2). Ann. Allergy, Asthma Immunol. 85, 9–21 (2000).
9. van Wilgenburg, B. et al. MAIT cells are activated during human viral infections. Nat. Commun. 7, 11653 (2016).
10. Vély, F. et al. Evidence of innate lymphoid cell redundancy in humans. Nat. Immunol. 17, 1291–1299 (2016).
11. Zuniga, E. I., Macal, M., Lewis, G. M. & Harker, J. A. Innate and Adaptive Immune Regulation During Chronic Viral Infections. Annu. Rev. Virol. 2, 573–597 (2015).
12. Lambert, L., Sagfors, A. M., Openshaw, P. J. M. & Culley, F. J. Immunity to RSV in early life. Front. Immunol. 5, 1–14 (2014).
13. Levy, O. Innate immunity of the newborn: Basic mechanisms and clinical correlates. Nat. Rev. Immunol. 7, 379–390 (2007).
14. Bianca Bennstein, S., Riccarda Manser, A., Weinhold, S., Scherenschlich, N. & Uhrberg, M. OMIP‐055: Characterization of Human Innate Lymphoid Cells from Neonatal and Peripheral Blood. Cytom. Part A 95, 427–430 (2019).
15. Costanzo, M. C., Creegan, M., Lal, K. G. & Eller, M. A. OMIP-027: Functional analysis of human natural killer cells. Cytom. Part A 87, 803–805 (2015).
16. Mahnke, Y. D., Beddall, M. H. & Roederer, M. OMIP-029: Human NK-cell phenotypization. Cytom. Part A 87, 986–988 (2015).
17. Nagakumar, P. et al. Type 2 innate lymphoid cells in induced sputum from children with severe asthma. J. Allergy Clin. Immunol. 137, 624–626 (2016).
18. Hilvering, B. et al. Synergistic activation of pro-inflammatory type-2 CD8+ T lymphocytes by lipid mediators in severe eosinophilic asthma. Mucosal Immunol. 11, 1408 – 419 (2018).
19. Wang, H. & Hogquist, K. A. How MAIT cells get their start. Nat. Immunol. 17, 1238–1240 (2016).
20. Ghazarian, L., Caillat-Zucman, S. & Houdouin, V. Mucosal-associated invariant T cell interactions with commensal and pathogenic bacteria: Potential role in antimicrobial immunity in the child. Front. Immunol. 8, 1–8 (2017).
21. Hammer Q, Romagnani C. OMIP-039: Detection and analysis of human adaptive NKG2C + natural killer cells. Cytom Part A. 91:997–1000 (2017).
22. Michaud A, Dardari R, Charrier E, Cordeiro P, Herblot S, Duval M. IL-7 enhances survival of human CD56bright NK cells. J Immunother. 33(4):382–90 (2010).
23. Wallrapp, A. et al. The neuropeptide NMU amplifies ILC2-driven allergic lung inflammation. Nature 549, 351–356 (2017).
24. Mahnke YD, Beddall MH, Roederer M. OMIP-019: Quantification of human γδT-cells, iNKT-cells, and hematopoietic precursors. Cytom Part A. 83(8):676–8 (2013).
25. Wistuba-Hamprecht K, Pawelec G, Derhovanessian E. OMIP-020: Phenotypic characterization of human γδ T-cells by multicolor flow cytometry. Cytom Part A. 85(6):522–4 (2014).
26. Bocsi J, Melzer S, Dähnert I, Tárnok A. OMIP-023: 10-Color, 13 antibody panel for in-depth phenotyping of human peripheral blood leukocytes. Cytom Part A. 85(9):781– (2014).
27. Pitoiset, F. et al. A standardized flow cytometry procedure for the monitoring of regulatory T cells in clinical trials. Cytom. Part B Clin. Cytom. 348–355 (2018).
28. Streitz, M. et al. Standardization of whole blood immune phenotype monitoring for clinical trials: panels and methods from the ONE study. Transplant. Res. 2, 17 (2013).
29. Masuda, A. et al. Role of Fc Receptors as a therapeutic target. Inflamm. Allergy Drug Targets 8, 80–6 (2009).
30. Nishimura, M., Mitsunaga, S. & Juji, T. Frozen-stored granulocytes can be used for an immunofluorescence test to detect granulocyte antibodies. Transfusion (2001).
31. Canonico, B. et al. Evaluation of leukocyte stabilisation in TransFix®-treated blood samples by flow cytometry and transmission electron microscopy. J. Immunol. Methods 295, 67–78 (2004).
32. Jozwik, A. et al. RSV-specific airway resident memory CD8+ T cells and differential disease severity after experimental human infection. Nat. Commun. 6, 1–15 (2015).
33. Thwaites, R. S. et al. Nasosorption as a minimally invasive sampling procedure: Mucosal viral load and inflammation in primary RSV bronchiolitis. J. Infect. Dis. 215, 1240–1244 (2017).


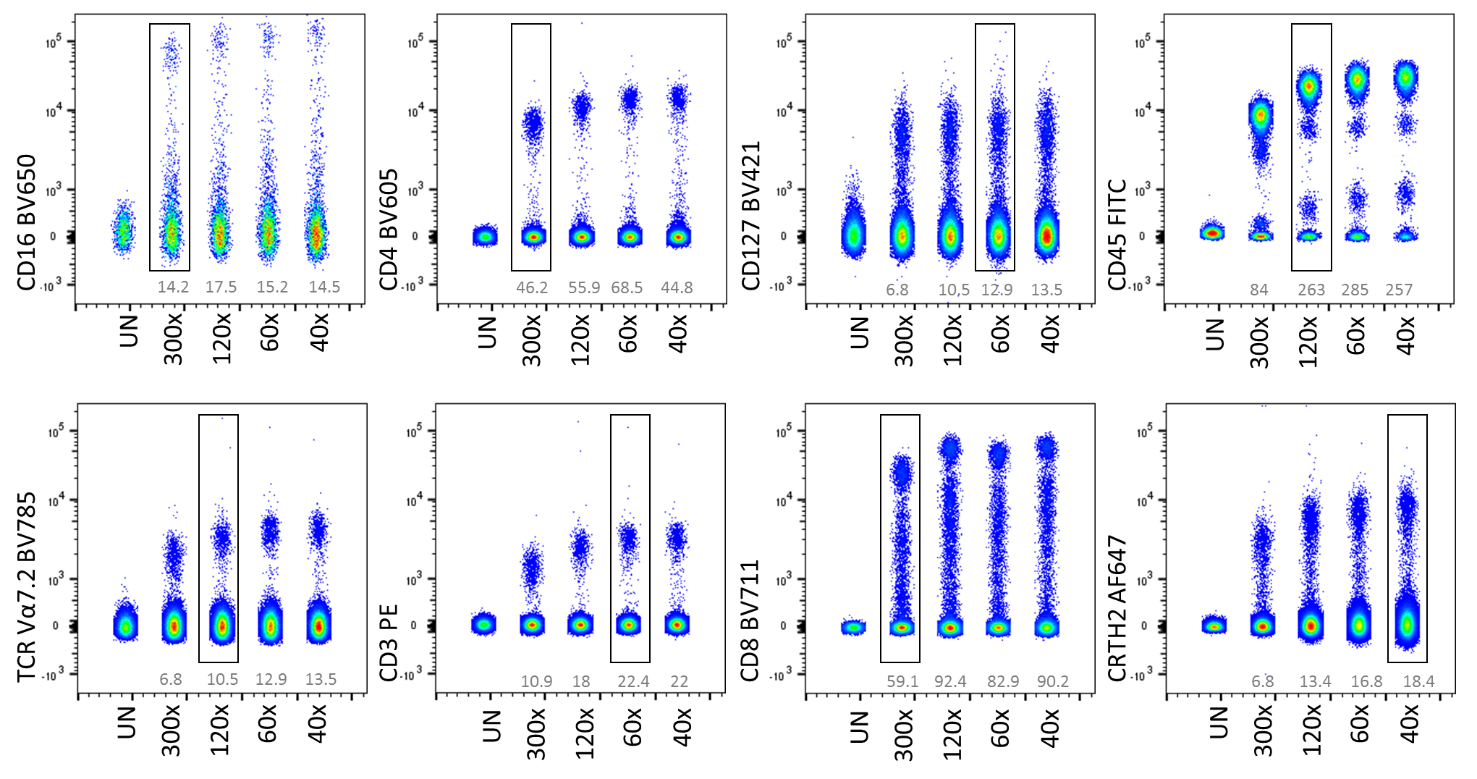

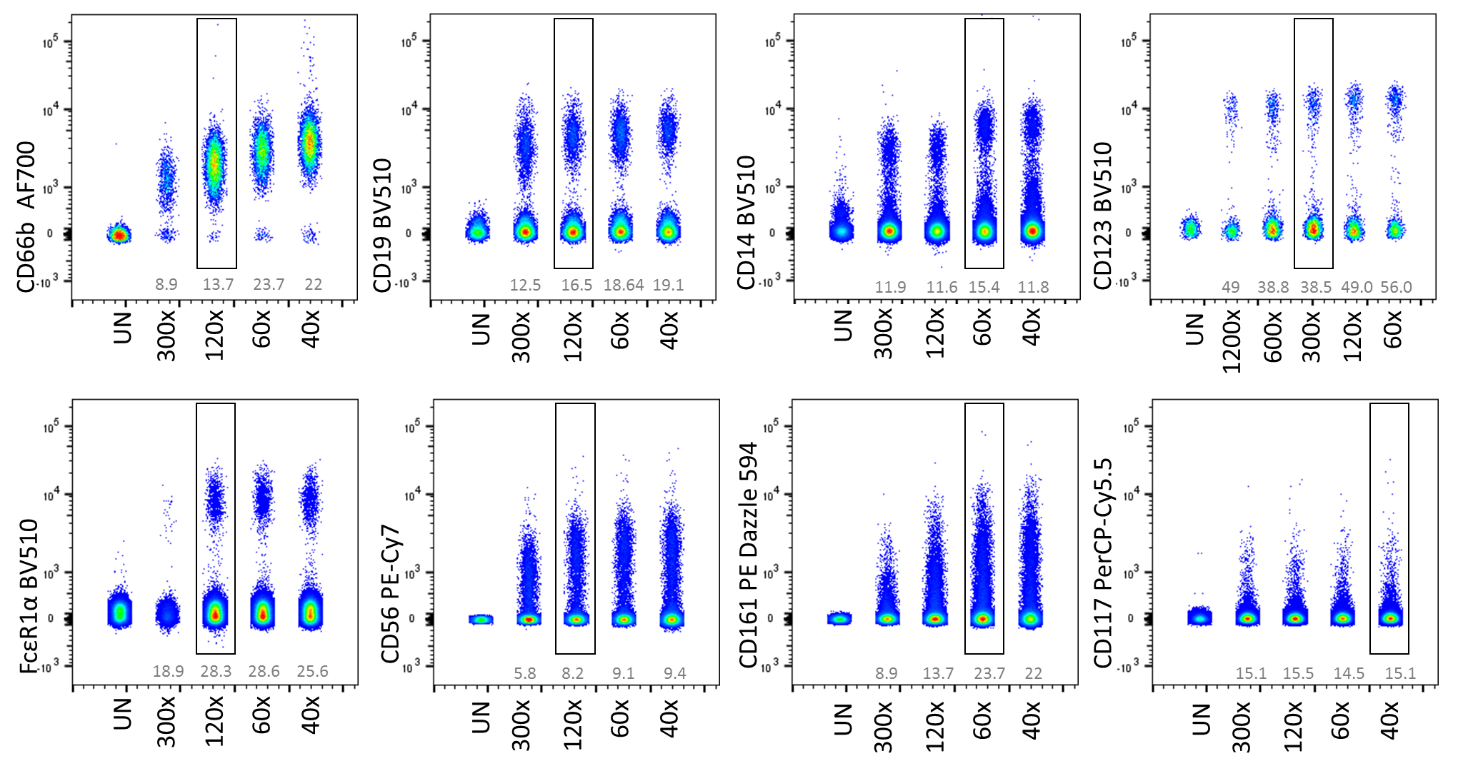
**Online Figure 1. Antibody titrations.** All antibodies were individually titrated on whole blood from healthy adult volunteers. Data is gated on the appropriate area of the FSC/SSC plot. The stain index is indicated for each dilution and the recommended dilution indicated.


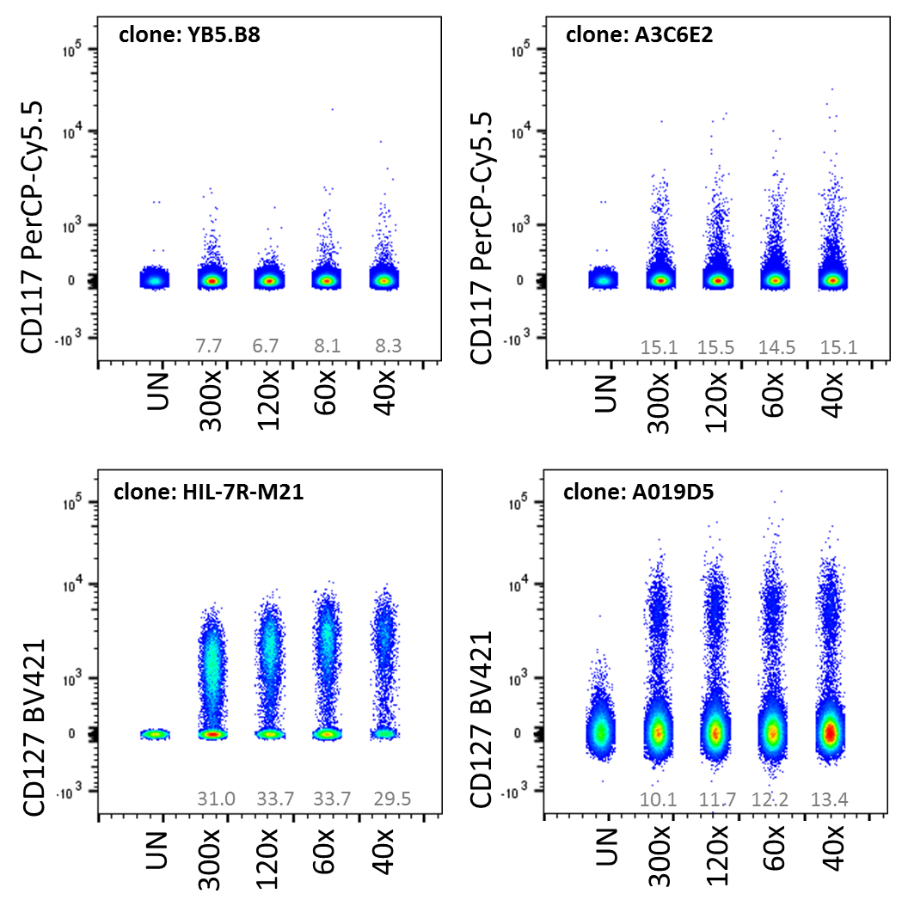


**Online Figure 2. Titration data for CD117 and CD127 antibodies.** Titration of different clones of CD117 (YB5.B8 and A3C6E2) and CD127 (HIL-7R-M21 and A019D5). Antibodies were individually titrated on whole blood from healthy adult volunteers. Data is gated on the appropriate area of the FSC/SSC plot. The stain index is indicated for each dilution.


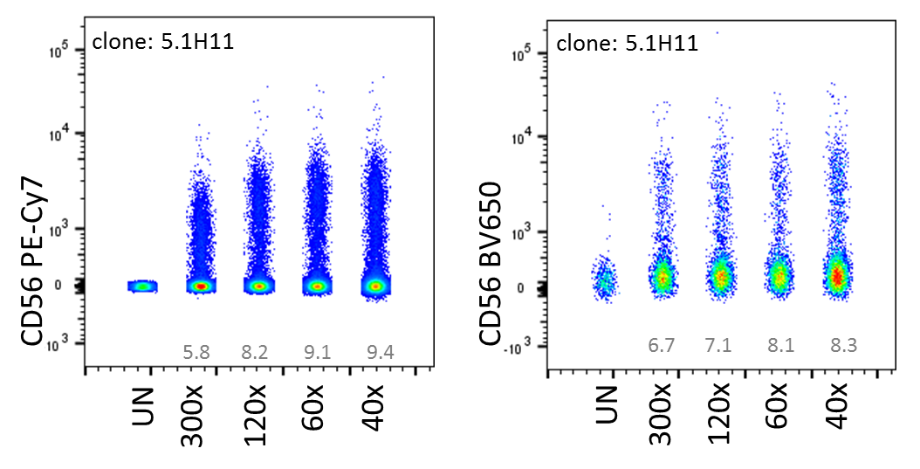


**Online Figure 3. Titration data for CD56 PE-Cy7 and CD56 BV650 clone 5.1H11.** Antibodies were individually titrated on whole blood from healthy adult volunteers. Data is gated on the appropriate area of the FSC/SSC plot. The stain index is indicated for each dilution.


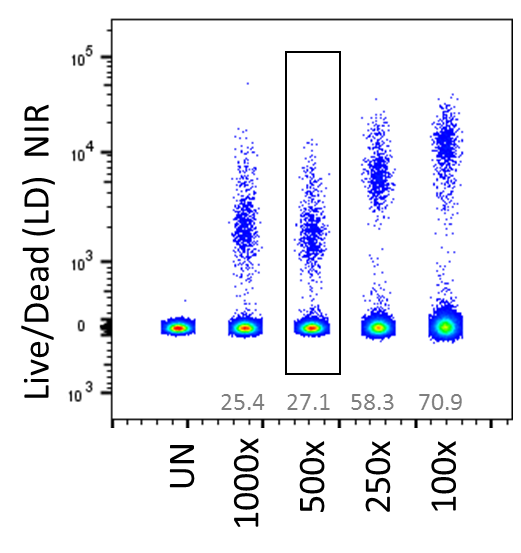


**Online Figure 4. Live/Dead reagent titration.** LIVE/DEAD™ Fixable Near-IR Dead Cell Stain Kit was titrated on whole blood from healthy adult volunteers according to the manufacturer’s instructions. The recommended dilution is indicated.

**
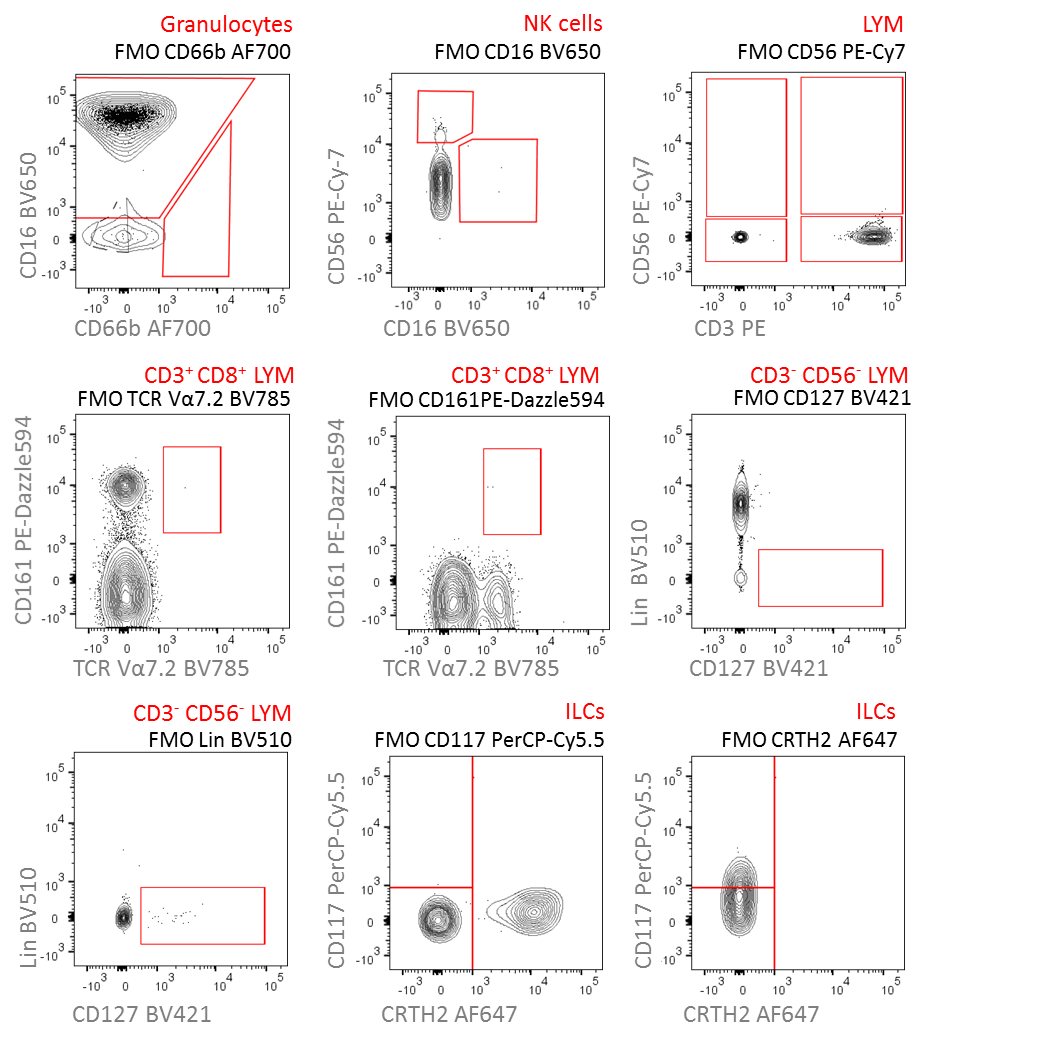
**

**Online Figure 5. Fluorescence Minus One (FMO) controls.** Staining was performed on adult whole blood. Each plot was gated on the cell type indicated in red.

**
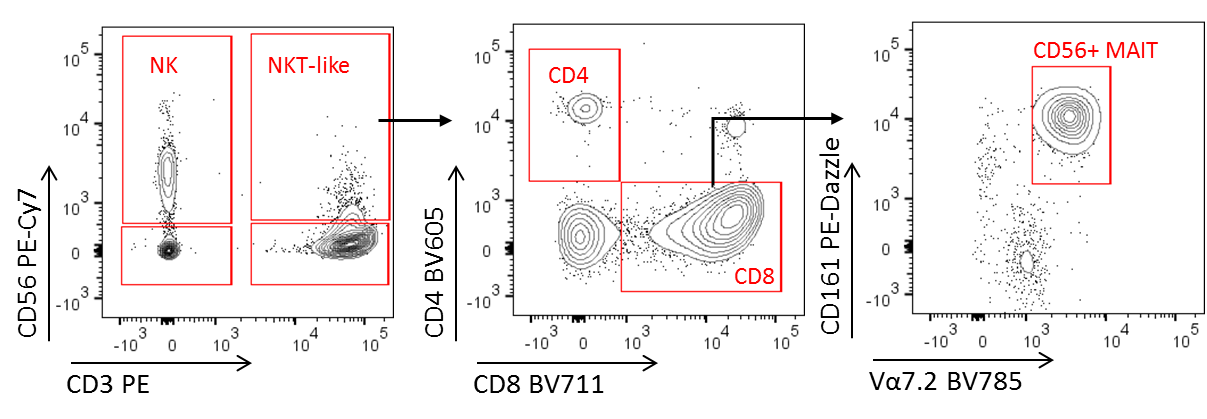
**

**Online Figure 6. Exemplified gating strategy for NKT-like cell subsets.** Using the OMIP, CD45^+^ CD56^+^ CD3^+^ lymphocytes are defined as NKT-like cells. Within this population, CD4^+^, CD8^+^ and CD56^+^ MAIT (CD8^+^ CD161^H^ Vα7.2^+^) sub-populations can be defined.

**
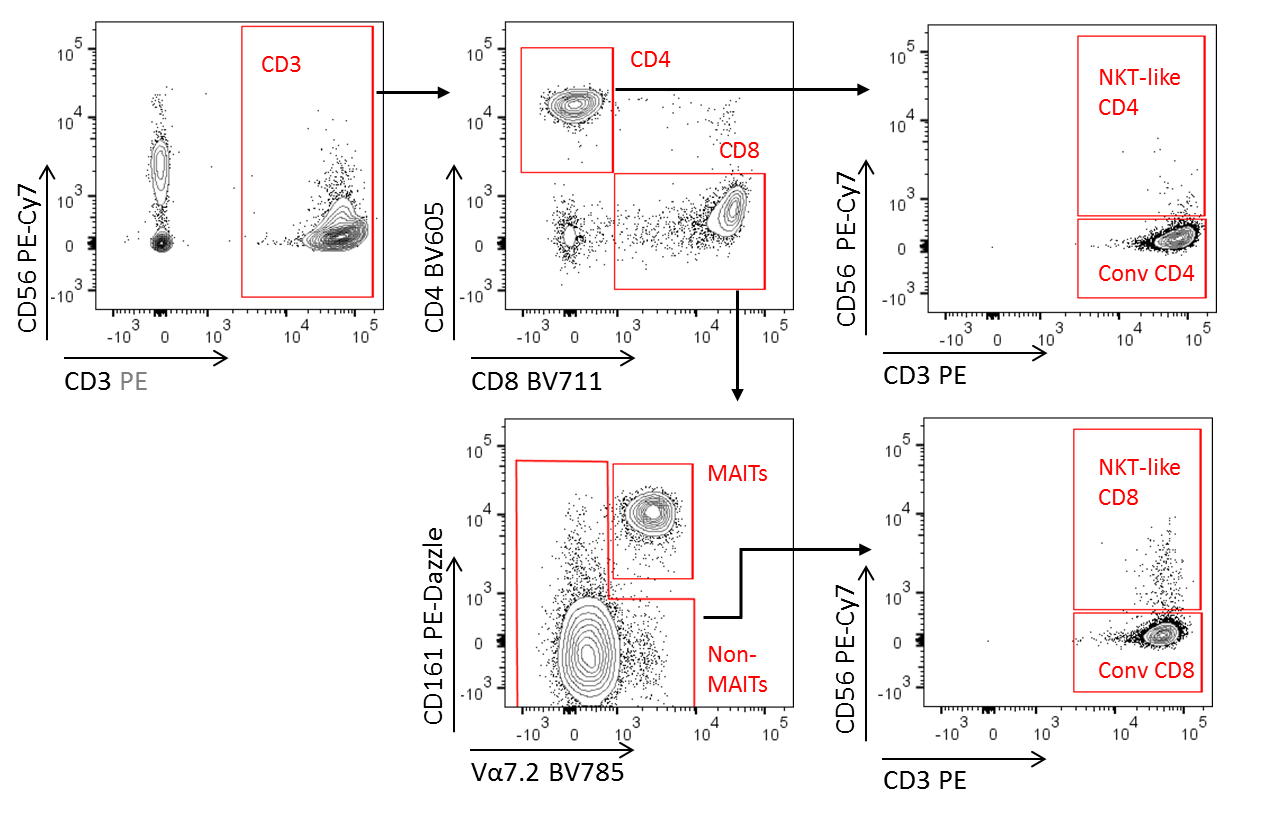
**

**Online Figure 7. Alternative gating strategy for identification of MAIT cells.** Using the OMIP, CD45^+^CD3^+^ lymphocytes are sub-divided into CD4^+^, CD8^+^ cells and total MAIT cells identified as CD161^H^ Vα7.2^+^ cells within the CD8^+^ population. Non-MAIT CD8^+^ cells can then be sub-divided into NKT-like cells and conventional CD8+ lymphocytes. Abbreviations used: Conv – conventional.

**
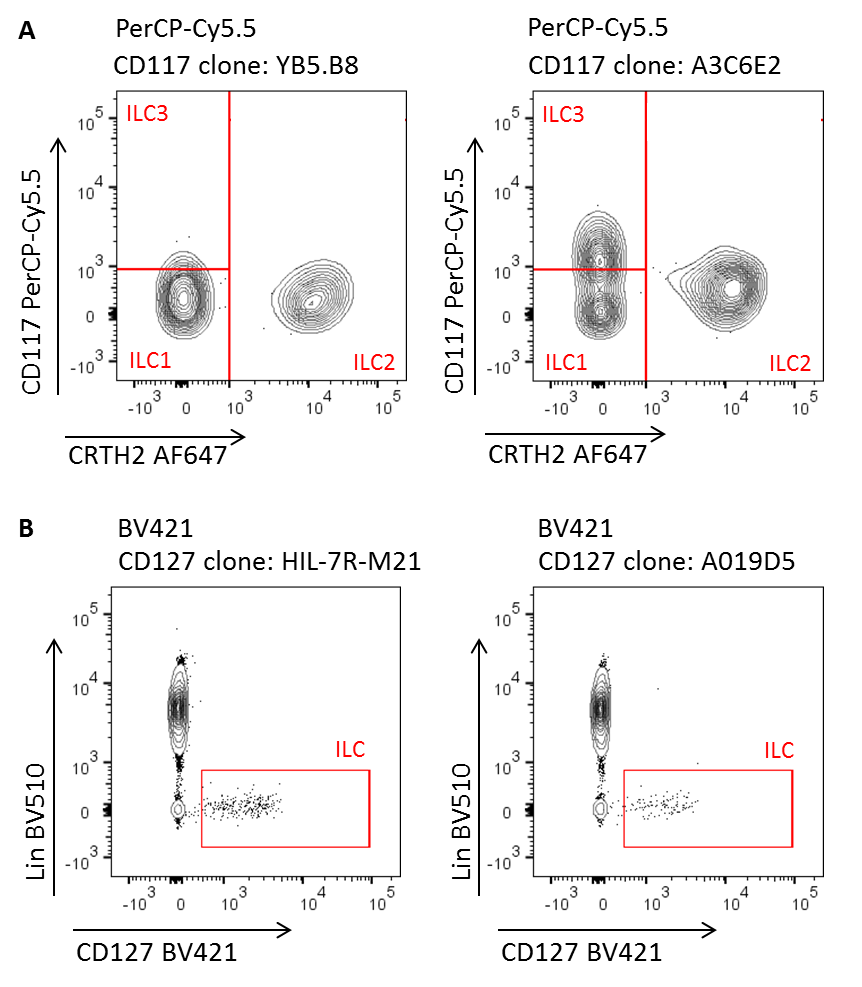
**

**Online Figure 8. Comparison of CD117 PerCP-Cy5.5 (clones YB5.B8 ad A3C6E2) and CD127 BV421 (clones HIL-7R-M21 and A019D5).** Whole blood was stained using the OMIP with different clones of anti-human CD117 (1:40) and anti-human CD127 (1:60). **(A)** For CD117 pictographs, ILCs were identified by gating as shown in Figure 1 Part A on CD45^+^CD56^-^CD3^-^Lin^-^CD127^+^ lymphocytes. **(B)** CD127 staining is shown after gating for CD45^+^CD56^-^CD3^-^ lymphocytes.


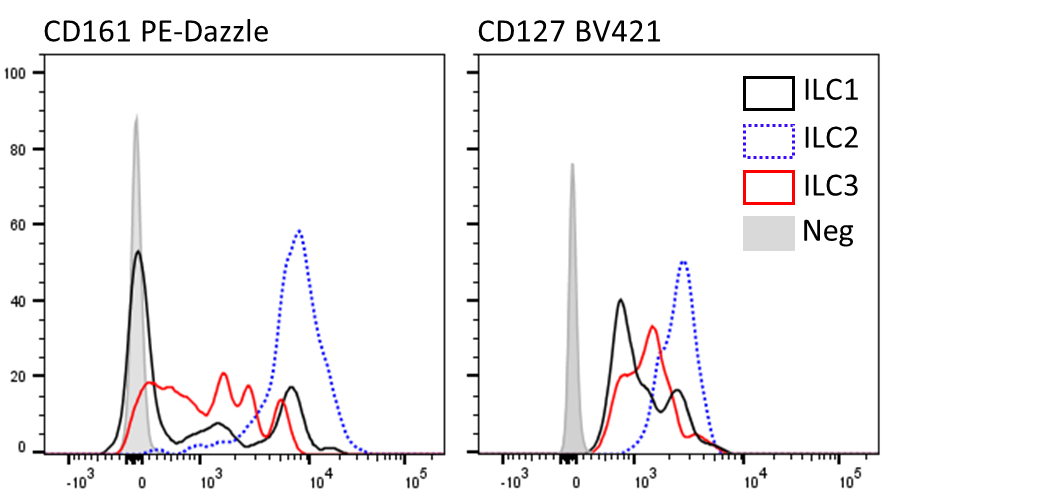


**Online Figure 9. Histograms of CD161 and CD127 expression on ILCs.** ILCs were defined using the following gating strategy: live, single, CD45^+^, lineage negative (CD3, CD14, CD19, CD56, CD123, FcεR1α)^-^ and CD127^+^ lymphocytes. Histograms show levels of expression of CD161 and CD127 on ILC subsets and FMO controls (Neg).


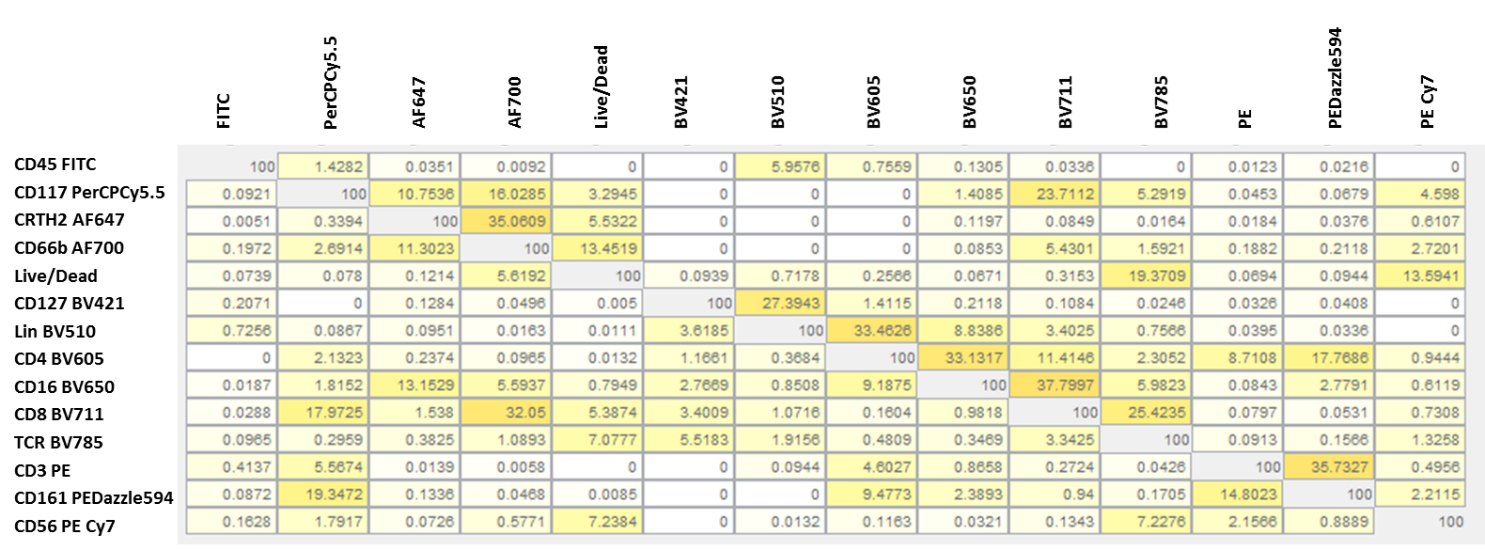


**Online Figure 10 Compensation matrix (based on the data shown in Figure 1, Part A)**

**
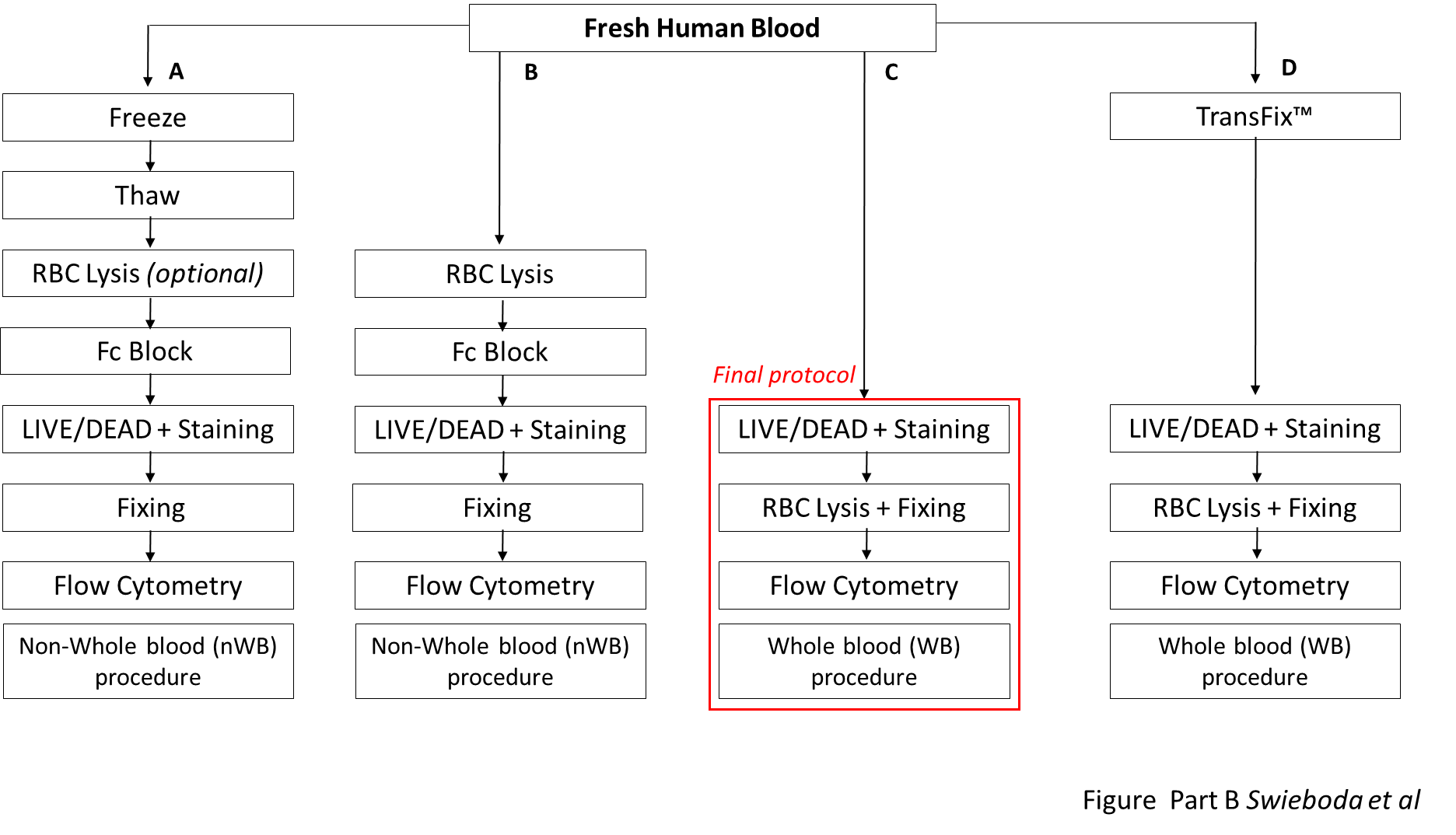
**

**Online Figure 11. Alternative strategies for blood sample processing tested during panel optimization.** Fresh blood was frozen and stained for flow cytometric analysis (**A**) or processed fresh with red blood cell lysis (RBC Lysis) before (**B**) or after (**C**) staining. Fresh whole blood was stabilized using TransFix™ reagent before staining (**D**). Non-whole blood (nWB) procedures involve RBC lysis or freezing before immunophenotyping. Abbreviation used: Fc – Fc receptor, RBC – red blood cells.


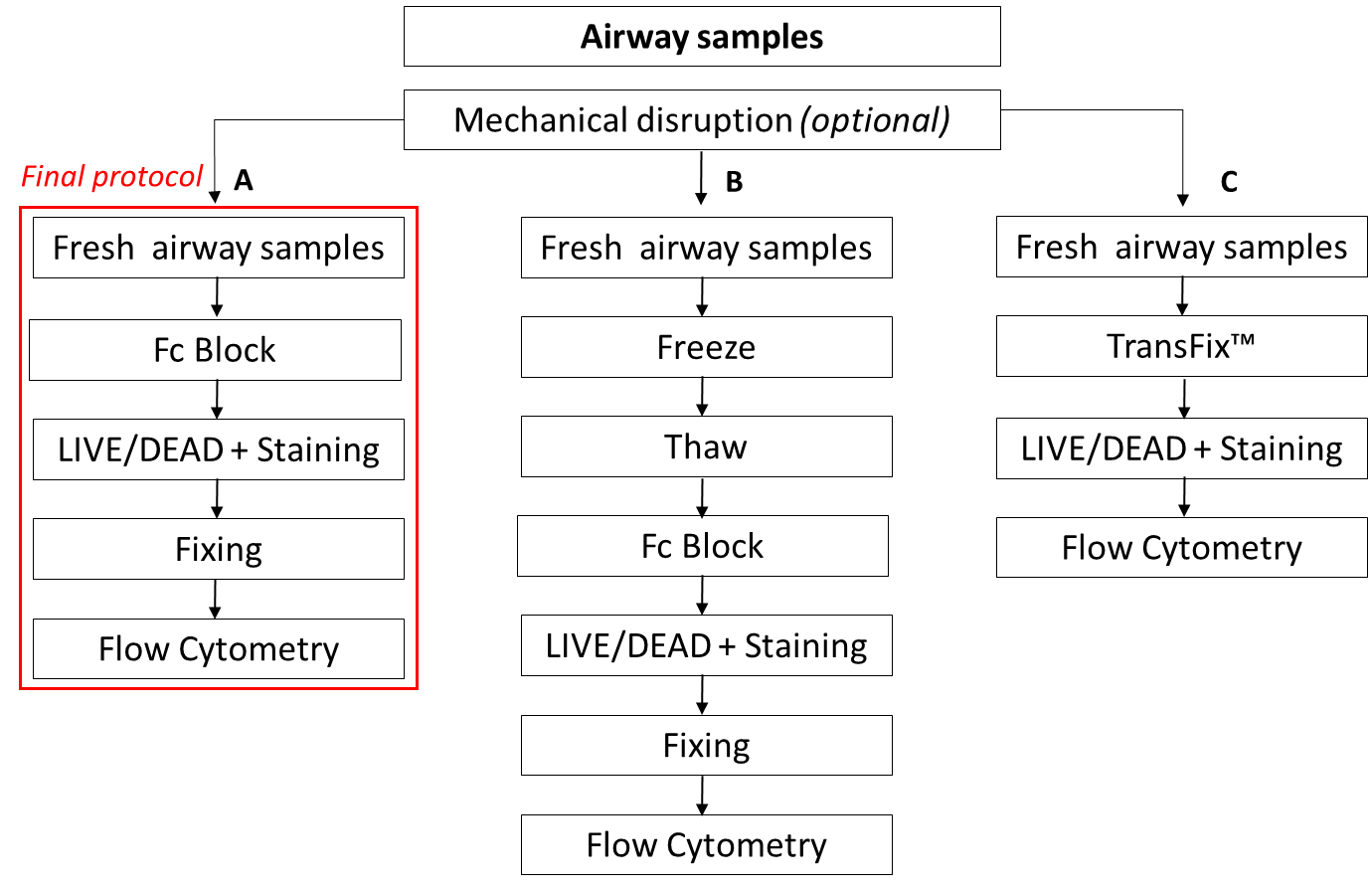


**Online Figure 12. Alternative strategies for airway sample processing tested during panel optimization.** Fresh (**A**), frozen (**B**) or fresh airway samples stabilized using TransFix™ reagent (**C**) were stained for flow cytometric analysis. Mechanical disruption with a syringe and a needle was performed when samples were mucous-rich.

| **NPA** |
| --- |
| 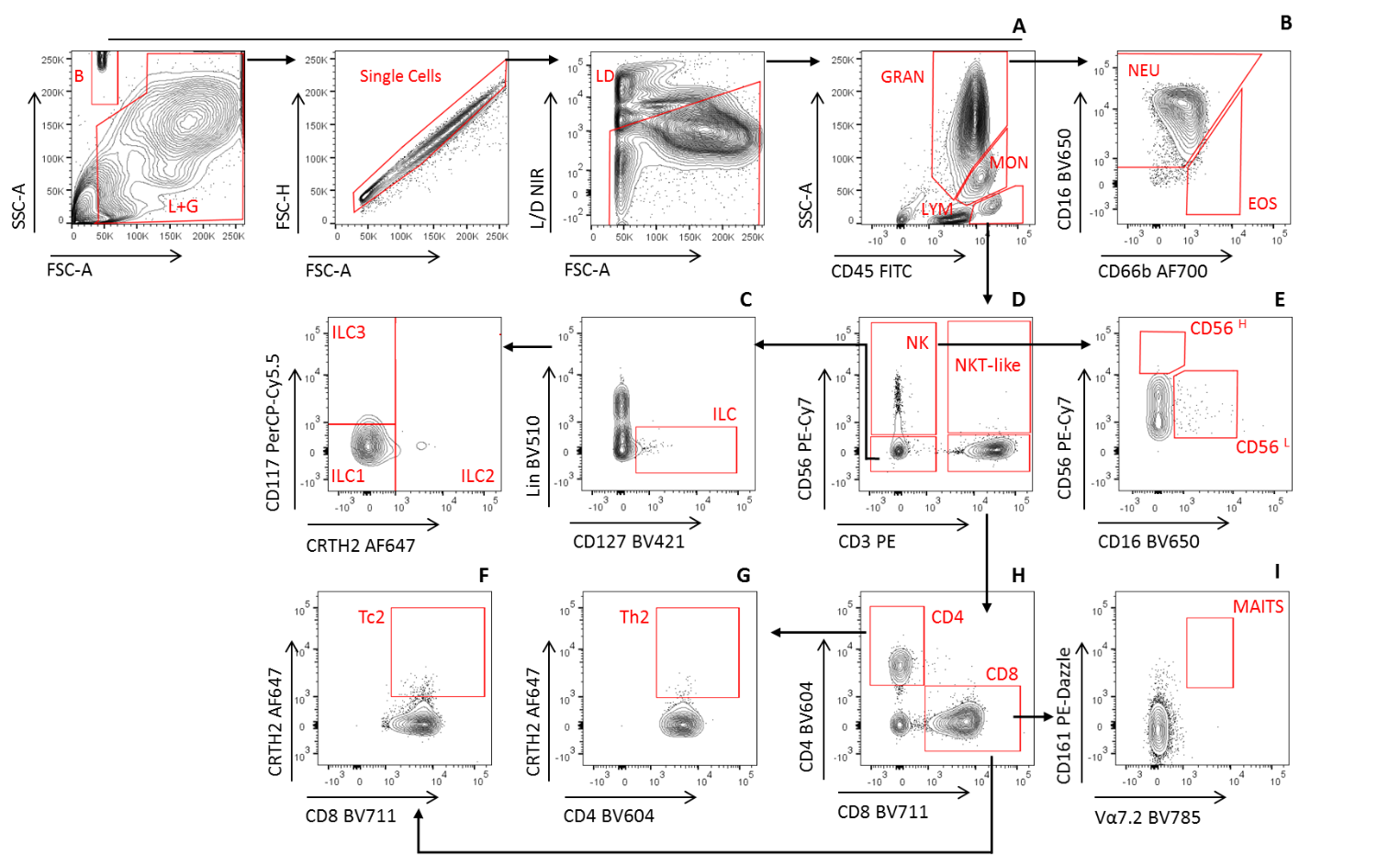 |
| **TA** |
| 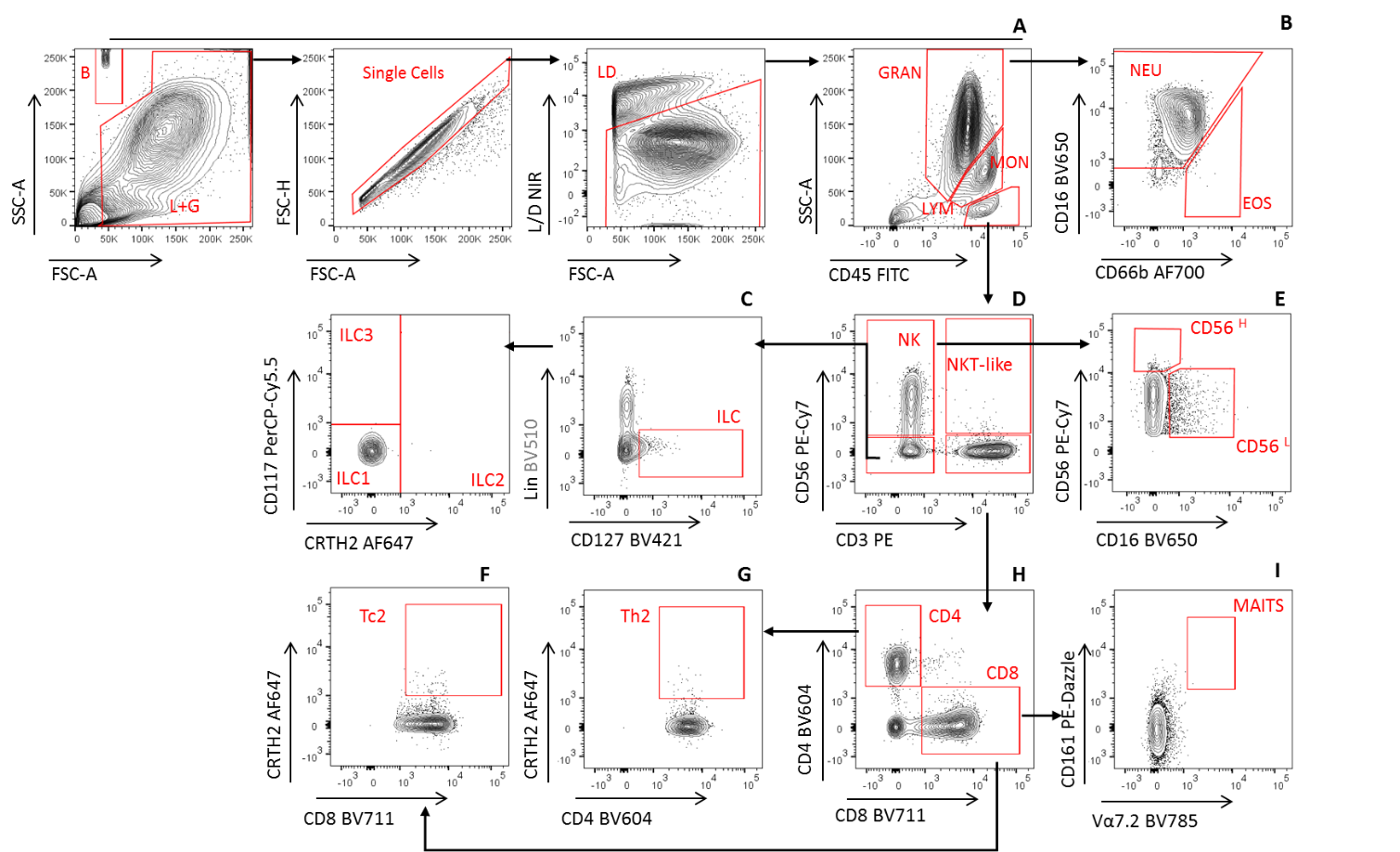 |

**Online Figure 13. Gating strategy for enumeration of lymphocytes and granulocytes in pediatric nasopharyngeal aspirate (NPA) and tracheal aspirate (TA).** Cells were delineated using the following gating strategy: FSC/SSC, single, live cells. Granulocytes, monocytes and lymphocytes were gated using FSC and CD45 **(A)**. Within the granulocytes, neutrophils were defined as CD16^H^CD66b^+/-^ and eosinophils as CD16^+/-^CD66b^+^ **(B)**. NK and NKT-like cells were gated within the lymphocyte gate as CD56^+^CD3^-^ and CD56^+^CD3^+^ cells, respectively **(D)**. NK cells were further subdivided into CD56^H^ and CD56^L^ populations according to the CD56 and CD16 cell markers **(E)**. T lymphocytes (CD3^+^CD56^-^) were segregated into CD4^+^ or CD8^+^ T cells **(H)**. Type-2 T cells were defined within the CD4^+^ and CD8^+^ populations using the CRTH2 surface marker **(F, G)**. MAIT cells are CD161^H^ Vα7.2^+^ and were gated from live, single, CD45^+^, CD56^-^, CD3^+^, CD8^+^ lymphocytes **(I)**. ILCs were defined as live, single, CD45^+^, lineage negative (CD3, CD14, CD19, CD56, CD123, FcεR1α)^-^ and CD127^+^ lymphocytes **(C)**; ILC1 were defined as CD117^−^CRTH2^−^, ILC2 as CRTH2^+^ CD117^int^ and ILC3 as CD117^+^CRTH2^−^ . Abbreviations used: B – counting beads, L+G – lymphocyte and granulocyte gate, LD – Live/Dead, GRAN – granulocytes, MON – monocytes, LYM – lymphocytes, NEU – neutrophils, EOS – eosinophils, NK – natural killer cells, NKT like – natural killer T –like cells.

**
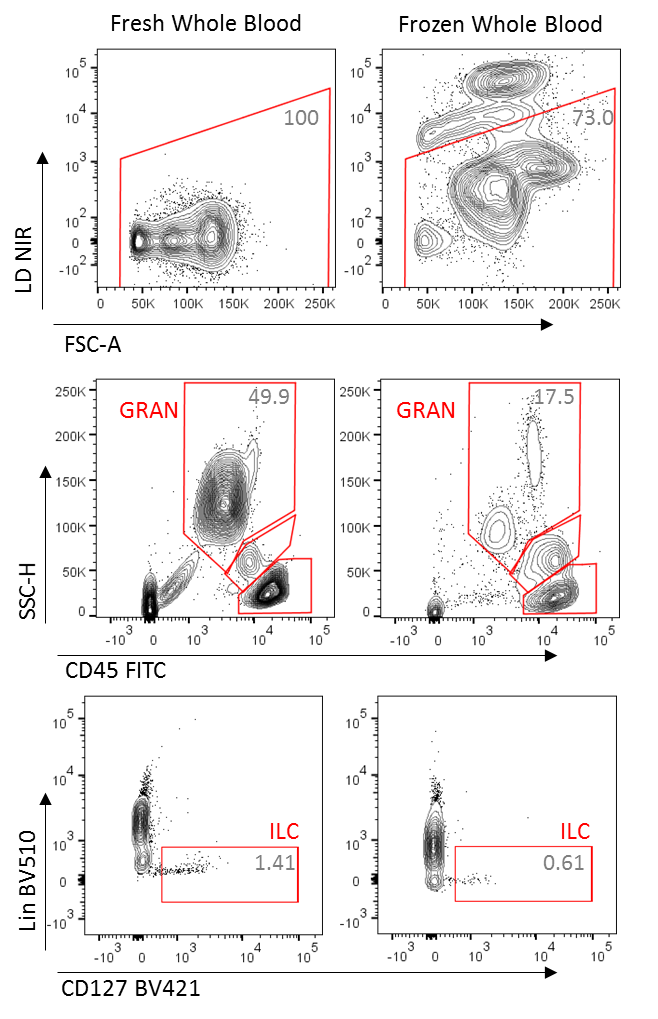
**

**Online Figure 14. Freezing of fresh whole blood and storage in liquid nitrogen reduces the total number of live cells and granulocytes upon recovery.** Fresh blood was stained directly with Live/Dead stain and antibodies, then red blood cell lysis and fixation performed (WB – whole blood) followed by analysis using flow cytometry. Freezing medium (90% FCS, 10% DMSO) was used for long – term storage in liquid nitrogen after controlled – rate freezing to -80^o^C in Nalgene™ Mr Frosty (FWB – frozen whole blood) prior to staining and analysis. Live, CD45 positive cells and ILCs are shown. Abbreviations used: GRAN – granulocytes, ILC – innate lymphoid cells.


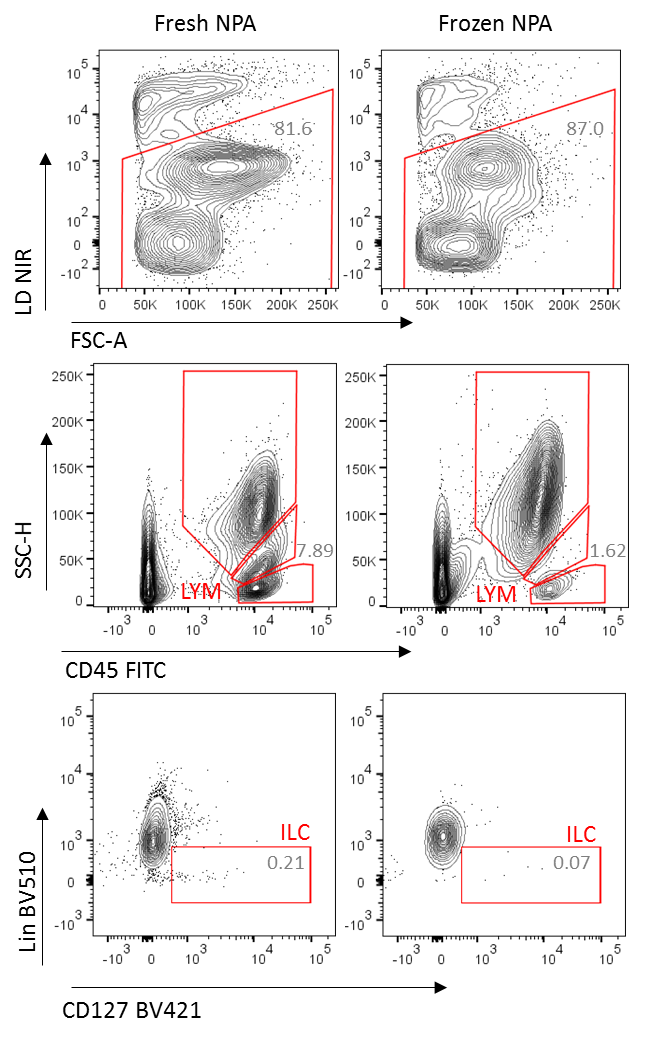


| **Online Figure 15. Comparison of the effect of different processing protocols on lymphocytes and ILCs in nasopharyngeal aspirate (NPA)**. Live cells were analyzed following staining and flow cytometry of NPA samples. The flow plots show the quantification of live, CD45^+^ lymphocytes and ILCs. |
| --- |
|  |
| 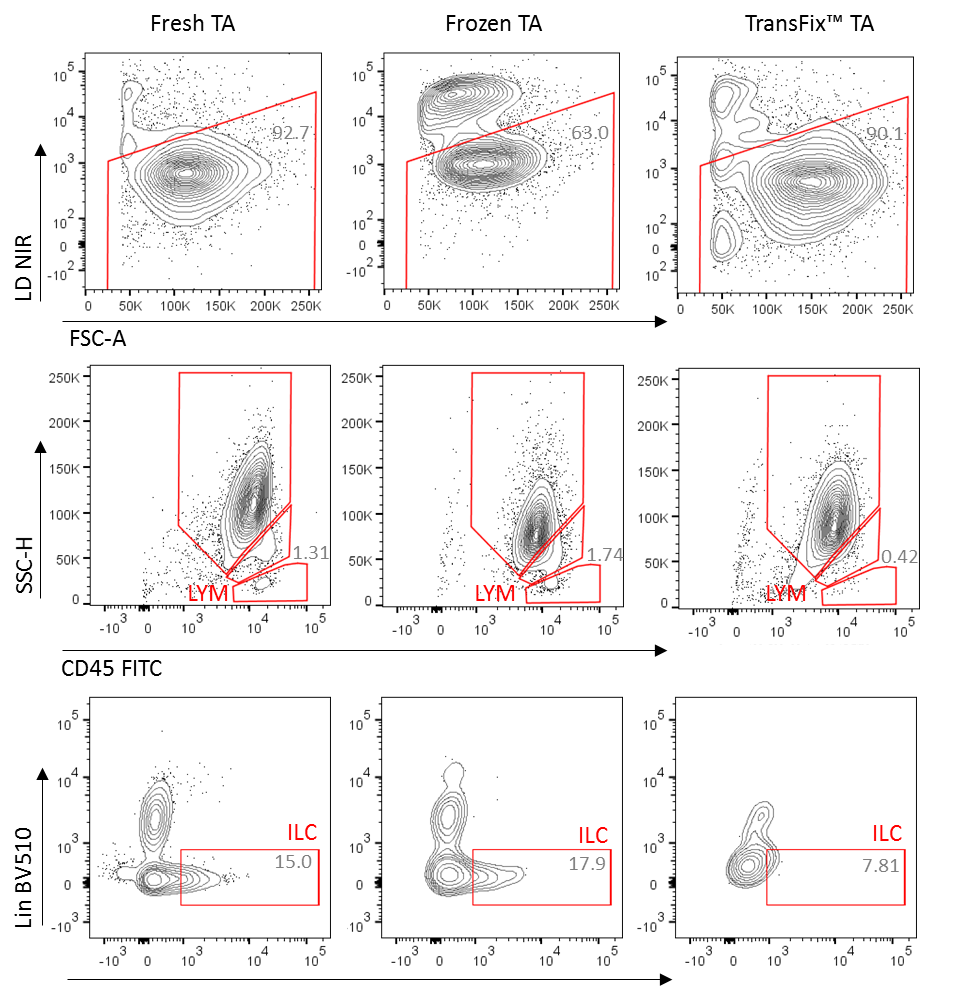 |
| **Online Figure 16. Comparison of different processing protocols on the identification of lymphocytes and ILC in a tracheal aspirate (TA) sample**. The flow plots show the quantification of live cells, CD45^+^ lymphocytes and ILCs when the TA sample was processed fresh, frozen or stabilized in TransFix prior to staining. |

**
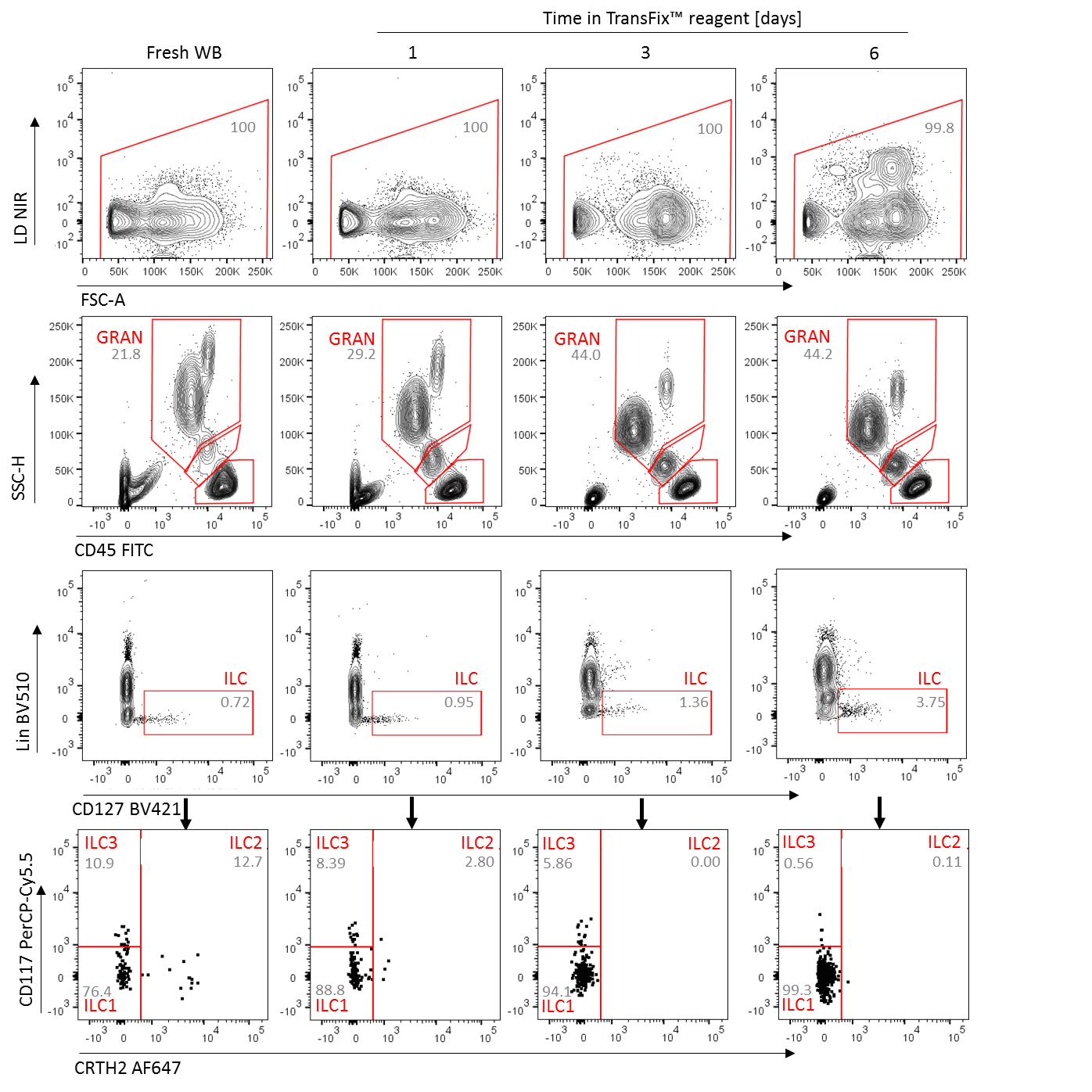
**

**Online Figure 17. Blood samples stabilized with Transfix™ show a time-dependent decrease in the brightness of CRTH2 staining on ILC2s.** Blood was preserved in Transfix™ reagent (1, 3 and 6 days) prior to staining and analysis and compared to fresh whole blood (WB). The impact of Transfix™ reagent on live cells, granulocytes (GRAN), innate lymphoid cells (ILCs) and ILC subsets was determined.

| **Laser Wavelength (nm)** | **Laser Power (mW)** | **Laser Type** | **Detector** | **Spectral range (nm)** | **Dichroic LP Filter (nm)** | **Band Pass (nm)** | **Fluorochrome** |
| --- | --- | --- | --- | --- | --- | --- | --- |
| Violet laser 405 | 100 | DPSS | 405-450/50 | 425/475 | - | 450/50 | BV421 |
|  |  |  | 405-525/50 | 500/550 | 475LP | 525/50 | BV510 |
|  |  |  | 405-605/12 | 599/611 | 595LP | 605/12 | BV605 |
|  |  |  | 405-655/8 | 651/659 | 635LP | 655/8 | BV650 |
|  |  |  | 405-710/50 | 690/730 | 690LP | 710/40 | BV711 |
|  |  |  | 405-780/60 | 750/810 | 735LP | 780/60 | BV785 |
| Blue laser 488 | 100 | DPSS | 488-530/30 | 515/545 | 505LP | 530/30 | FITC |
|  |  |  | 488-710/50 | 685/735 | 685LP | 710/50 | PerCP Cy5.5 |
| Yellow/Green  561 | 100 | DPSS | 561-582/15 | 575/590 | 570LP | 582/15 | PE |
|  |  |  | 561-620/10 | 615/625 | 600LP | 620/10 | PE Dazzle 594 |
|  |  |  | 561-780/60 | 750/810 | 750LP | 780/60 | PECy7 |
| Red laser 640 | 70 | DPSS | 633-670/14 | 663/677 | - | 670/14 | AF647 |
|  |  |  | 633-720/40 | 700/740 | 710LP | 720/40 | AF700 |
|  |  |  | 633-780/60 | 750/810 | 750LP | 780/60 | Near-IR fluorescent dye |

**Online Table 1. Instrument and configuration settings.** The panel was developed and optimized on an LSRFortessa™ flow cytometer using the instrument configuration listed.

| **Antigen** | **Clone** | **Fluorochrome** | **Product code** | **Company** | **Species** | **Isotype** | **Dilution** |
| --- | --- | --- | --- | --- | --- | --- | --- |
| CD127 (IL-7Rα) | A019D5 | BV421 | 351309 | BioLegend | mouse | IgG1, κ | 1:60 |
| CD14 | 63D3 | BV510 | 367123 | BioLegend | mouse | IgG1, κ | 1:60 |
| CD19 | HIB19 | BV510 | 302241 | BioLegend | mouse | IgG1, κ | 1:120 |
| FcεRIα | AER-37, (CRA-1) | BV510 | 334626 | BioLegend | mouse | IgG1, κ | 1:120 |
| CD123 | 6H6 | BV510 | 306021 | BioLegend | mouse | IgG1, κ | 1:300 |
| CD4 | RPA-T4 | BV605 | 300555 | BioLegend | mouse | IgG1, κ | 1:300 |
| CD16 | 3G8 | BV650 | 302041 | BioLegend | mouse | IgG1, κ | 1:300 |
| CD8 | SK1 | BV711 | 344733 | BioLegend | mouse | IgG1, κ | 1:300 |
| TCR Vα7.2 | 3C10 | BV785 | 351721 | BioLegend | mouse | IgG1, κ | 1:120 |
| CD45 | HI30 | FITC | 351721 | BioLegend | mouse | IgG1, κ | 1:300 |
| CD117 (c-kit) | A3C6E2 | PerCP-Cy5.5 | 323416 | BioLegend | mouse | IgG1, κ | 1:40 |
| CD3 | OKT3 | PE | 317307 | BioLegend | mouse | IgG2a | 1:60 |
| CD161 | HP-3G10 | PE-Dazzle | 339939 | BioLegend | mouse | IgG1, κ | 1:60 |
| CD56 (NCAM) | 5.1H11 | PE-Cy7 | 362509 | BioLegend | mouse | IgG1, κ | 1:120 |
| CD294 (CRTH2) | BM16 | AF647 | 350103 | BioLegend | rat | IgG2a | 1:40 |
| CD66b | G10F5 | AF700 | 305113 | BioLegend | mouse | IgM, κ | 1:120 |
| Viability |  | Near IR-fluorescent reactive dye | L10119 | Thermofisher |  |  | 1:500 |

**Online Table 2. Reagents used in the final panel with dilutions and manufacturer’s information.**

| **Reagents** | **Catalogue Number** | **Company** | **Country** |
| --- | --- | --- | --- |
| AbC™ Total Antibody Compensation Bead Kit | A10497 | Thermofisher | UK |
| ArC™ Amine Reactive Compensation Bead Kit | A10346 | Invitrogen | UK |
| Antibodies listed in **Table 2** (**Cytometry Part B**) | - | - | - |
| CD117 PerCP-Cy5.5 (clone YB5.B8) | 562094 | BD Bioscience | UK |
| CD127 BV421 (clone HIL-7R-M21) | 562437 | BD Bioscience | UK |
| CD56 BV650 (clone 5.1H11) | 362532 | BioLegend | UK |
| Cell staining buffer | 420201 | BioLegend | UK |
| CountBright™ Absolute Counting Beads | 100446 | Thermofisher | UK |
| CS&T Research Beads | 655051 | BD Bioscience | UK |
| DMSO | 67-68-5 | Sigma-Aldrich | UK |
| FCS | F9665 | Sigma-Aldrich | UK |
| Fixation Buffer | 420801 | BioLegend | UK |
| Human TruStain FcX™ - Fc Block | 422302 | BioLegend | UK |
| LIVE/DEAD™ Fixable Near-IR Dead Cell Stain Kit | L10119 | Thermofisher | UK |
| Lymphopure | 426201 | BioLegend | UK |
| Multicounting10™ Cell counting chamber slides | MC100 | Immune Systems | UK |
| PBS | D5537 | Sigma-Aldrich | UK |
| RBC Lysis Buffer (10x) | 420301 | BioLegend | UK |
| RBC Lysis/Fixation Solution (10x) | 422401 | BioLegend | UK |
| TransFix™ | TFB-01-10 | CalTag | UK |
| Trypan Blue | T10282 | Thermofisher | UK |

**Online Table 3. Reagents used.**
