# Supplementary material for: OMIP‐062: A 14‐Color, 16‐Antibody Panel for Immunophenotyping Human Innate Lymphoid, Myeloid and T Cells in Small Volumes of Whole Blood and Pediatric Airway Samples
Source: Cytometry A. 2019 Oct 21;95(12):1231–5. doi: 10.1002/cyto.a.23907 (PMC6972618; doi:10.1002/cyto.a.23907)
Supplement: Supplementary file 2 — MIFlowCyt: MIFlowCyt‐Compliant Items [file CYTO-95-1231-s002.doc]

**Cytometry Part A**

**Author Checklist: MIFlowCyt-Compliant Items**

| **Requirement** | **Please Include Requested Information** |
| --- | --- |
| 1.1. Purpose | A 14-color, 16-antibody panel for immunophenotyping human innate lymphoid, myeloid and T cells in small volumes of whole blood and pediatric airway samples |
| 1.2. Keywords | T cells, Innate Lymphoid Cells (ILCs), NK cells, NKT cells, NKT-like cells, myeloid cells, granulocytes, mucosal-associated invariant T cells (MAIT cells), innate responses, type-2 responses, human whole blood immunophenotyping, respiratory, airway, pediatric. |
| 1.3. Experiment variables | Reagent concentration, cell subsets analyzed, different sample types |
| 1.4. Organization name and address | Imperial College London  National Heart and Lung Institute  Faculty of Medicine  St. Mary’s Campus  London  W2 1PG |
| 1.5. Primary contact name and email address | Fiona J. Culley  e-mail: [f.culley@imperial.ac.uk](mailto:f.culley@imperial.ac.uk) |
| 1.6. Date or time period of experiment | The panel was optimized between 11/2017 and 06/2018 |
| 1.7. Conclusions | Flow cytometry panel was designed for enumeration of all major members of innate lymphoid cell family (ILC1s including NK cells, ILC2s and ILC3s) in a small amount of whole blood and airway samples |
| 1.8. Quality control measures | Routine instrument Quality Check-up; day-to-day differences were calibrated by CST (cytometry setting and tracking) beads and the cytometry was compensated using compensation beads and PMT voltages were set up accordingly; CountBright™ were added and bead count and cells count was determined using the manufacturer’s instruction; Live/Dead control; fluorescence minus one (FMO) controls were utilized; using different types of samples; |
| 2.1.1.1. (2.1.2.1., 2.1.3.1.) Sample description | Fresh whole peripheral blood, TransFix™ stabilized whole blood, fresh and frozen PBMCs, airway samples, such as: tracheal aspirates (TA) and nasopharyngeal aspirates (NPA) were obtained from children (< 2 years of age) admitted to pediatric intensive care unit (PICU). |
| 2.1.1.2. Biological sample source description | Blood samples were obtained from healthy adults and healthy or bronchiolitic children, cord blood samples were taken after a C-section; All airways samples (NPA and TA) |
| 2.1.1.3. Biological sample source organism description | Human (Homo Sapiens) |
| 2.1.2.2. Environmental sample location | N/A |
| 2.3. Sample treatment description | Whole blood was stabilized during the optimization process or processed freshly. Whole blood freezing was performed as well; PBMCs were processes fresh or frozen; NPA and TA cells processed fresh, frozen or post stabilizing with TransFix™ |
| 2.4. Fluorescence reagent(s) description | | **Antibody** | **Fluorochrome** | **Ab Clone** | **Purpose** | | --- | --- | --- | --- | | CD127 (IL-7Rα) | BV421 | A019D5 | ILCs | | CD14* | BV510 | 63D3 | Lineage | | CD19* | BV510 | HIB19 | Lineage | | FcεRIα* | BV510 | AER-37 (CRA-1) | Lineage | | CD123* | BV510 | 6H6 | Lineage | | CD4 | BV605 | RPA-T4 | CD4^+^ T cells | | CD16 | BV650 | 3G8 | NK cells/neutrophils | | CD8 | BV711 | SK1 | CD8^+^ T cells | | TCR Vα7.2 | BV785 | 3C10 | MAIT cells | | CD45 | FITC | HI30 | Leukocytes | | CD117 (c-kit) | PerCP-Cy5.5 | A3C6E2 | ILC3 | | CD3** | PE | OKT3 | T cells | | CD161 | PE-Dazzle | HP-3G10 | MAIT cells | | CD56 (NCAM)** | PE-Cy7 | 5.1H11 | NK/NKT-like cells | | CD294 (CRTH2) | AF647 | BM16 | ILC2/Th2/Tc2 subsets | | CD66b | AF700 | G10F5 | Eosinophils | | Live/Dead | Near IR-fluorescent reactive dye | n/a | Viability |   **Antibodies used in the optimised multicolour immunofluorescence panel (OMIP).**  *Lineage cocktail includes the following antibodies: CD14, CD19, CD123, FcεR1α.  **Antibodies used to define the lineage negative population, but not part of the lineage cocktail. |
| 3.1. Instrument manufacturer | BD Bioscience |
| 3.2. Instrument model | 5-Laser-LSRFortessa™ |
| 3.3. Instrument configuration and settings | | **Laser Wavelength (nm)** | **Laser Power (mW)** | **Laser Type** | **Detector** | **Spectral range (nm)** | **Dichroic LP Filter (nm)** | **Band Pass (nm)** | **Fluorochrome** | | --- | --- | --- | --- | --- | --- | --- | --- | | Violet laser 405 | 100 | DPSS | 405-450/50 | 425/475 | - | 450/50 | BV421 | | 405-525/50 | 500/550 | 475LP | 525/50 | BV510 | | 405-605/12 | 599/611 | 595LP | 605/12 | BV605 | | 405-655/8 | 651/659 | 635LP | 655/8 | BV650 | | 405-710/50 | 690/730 | 690LP | 710/40 | BV711 | | 405-780/60 | 750/810 | 735LP | 780/60 | BV785 | | Blue laser 488 | 100 | DPSS | 488-530/30 | 515/545 | 505LP | 530/30 | FITC | | 488-710/50 | 685/735 | 685LP | 710/50 | PerCP Cy5.5 | | Yellow/Green  561 | 100 | DPSS | 561-582/15 | 575/590 | 570LP | 582/15 | PE | | 561-620/10 | 615/625 | 600LP | 620/10 | PE Dazzle 594 | | 561-780/60 | 750/810 | 750LP | 780/60 | PECy7 | | Red laser 640 | 70 | DPSS | 633-670/14 | 663/677 | - | 670/14 | AF647 | | 633-720/40 | 700/740 | 710LP | 720/40 | AF700 | | 633-780/60 | 750/810 | 750LP | 780/60 | Near-IR fluorescent dye | |
| 4.1. List-mode data files | *We recommend all authors to submit their data files to [http://flowrepository.org](http://flowrepository.org/) and to make them available for the peer-review process. If you have done so, please let us know by inserting the following codes (replace the red text):  1) The link for peer-review process:  http://flowrepository.org/id/RvFrxxxxxx (FR-FCM-Z28F). This link will only be shared with reviewers of your manuscript.  2) The repository identifier:  http://flowrepository.org/id/FR-FCM-xxxx (FR-FCM-Z28F). This link will be made publicly accessible after the paper is published. |
| 4.2. Compensation description | Automated compensation with FloJoV10 software (FlowJo, USA) |
| 4.3. Data transformation details | Bi-axial gating strategy |
| 4.4.1. Gate description | Files are not gated |
| 4.4.2. Gate statistics | Refer to the Figure 1 in the Part A of OMIP for the gating strategy |
| 4.4.3. Gate boundaries | Refer to the Supplementary online materials for details of setting gate boundaries |

**Notes**

Feel free to use more space than allocated.

You can embed graphics/figures in this document, if needed.

Please make sure to save the document in Microsoft Word version 2003 or older, before uploading to ScholarOne Manuscripts. When uploading this checklist to ScholarOne Manuscripts, please choose the “Supplementary Material for Review” category.

Please note that if your paper is accepted, the checklist will be published as an Online Supporting Information.

For any questions, please contact the Cytometry Part A editorial office at [Cytometrya@wiley.com](mailto:Cytometrya@wiley.com).
